# FieldML, a proposed open standard for the Physiome project for mathematical model representation

**DOI:** 10.1007/s11517-013-1097-7

**Published:** 2013-07-31

**Authors:** Randall D. Britten, G. Richard Christie, Caton Little, Andrew K. Miller, Chris Bradley, Alan Wu, Tommy Yu, Peter Hunter, Poul Nielsen

**Affiliations:** University of Auckland, Auckland, New Zealand

**Keywords:** Science technology, Life sciences biomedicine, Biochemical research methods, Mathematical computational biology, Mathematical models, FieldML, CellML

## Abstract

The FieldML project has made significant progress towards the goal of addressing the need to have open standards and open source software for representing finite element method (FEM) models and, more generally, multivariate field models, such as many of the models that are core to the euHeart project and the Physiome project. FieldML version 0.5 is the most recently released format from the FieldML project. It is an XML format that already has sufficient capability to represent the majority of euHeart’s explicit models such as the anatomical FEM models and simulation solution fields. The details of FieldML version 0.5 are presented, as well as its limitations and some discussion of the progress being made to address these limitations.

**Electronic supplementary material:**

The online version of this article (doi:10.1007/s11517-013-1097-7) contains supplementary material, which is available to authorized users.

## Introduction

CellML [2, 9] and FieldML [7] are open standards for declaratively representing mathematical models to facilitate model interchange and are primarily focussed on the needs of Physiome projects such as euHeart,^1^ a cardiac modelling project with a strong focus on clinical applications. We define a mathematical model to be a formulation that represents the state of a real-world system mathematically in such a way that the model can be used to make predictions about the real-world system by computing the state of the system based on the input parameters to the model. Functional models usually make predictions about dynamic systems. Geometric models vastly reduce the number of parameters when compared to the number of parameters required to record all the exact measurements of the real-world object. A distinction is made between implicit and explicit models. Explicit models are represented by plain numerical data and closed-form algebraic expressions, as well as certain functions commonly available in standard software math libraries, such as trigonometric functions, exponential, logarithm and so on. Implicit models include expressions that usually require the application of computational numerical solvers in order to evaluate, for example, systems of ordinary differential equations (ODEs) and partial differential equations (PDEs).

The Physiome Model Repository (PMR) software [41] provides a web repository for making models, based on these standards and other formats, easily accessible. Furthermore, PMR provides a collaboration workspace. The euHeart project aims to develop models, modelling standards and the related technologies that serve the goal of bridging the gap between cardiac modelling for research and the clinical application of individualised cardiac modelling. As part of the euHeart project, extensive design work has been done on the FieldML format and its API, and features for supporting FieldML in the PMR software.

The high-level goals of FieldML are to:

- Enable sufficient expressive power to represent fields pertinent to anatomical and physiological modelling;
- Allow models to contain sufficient information so as to be largely self-documenting;
- Represent multiscale Physiome models of anatomy and structure at scales from organism to cellular and molecular;
- Represent data in a way that is efficient in terms of computational costs such as disk space and data access times, making it possible to cope with large models;
- Be extensible, enabling future applications in areas not currently foreseen;
- Be simple enough to facilitate robust and simple implementation;
- Provide open source technology to achieve the above goals.

The goals of CellML have much in common, but CellML focusses on time-varying lumped parameter models. Although the format can express a wider range of models, most CellML software shares the focus on time-varying lumped parameter models [13, 29, 34]. CellML focusses on the algebraic and differential mathematical equations of the model, whereas currently, FieldML focusses both on describing fields over multiple discrete indices, through reference to sparsely or densely packed^2^ massive bulk numerical data, and on describing multivariate fields with some or all continuous variables, by defining finite element interpolations using the discrete data or by other interpolation methods or more general methods.

Computer-readable model representation formats for Physiome models such as those used by the euHeart project need to support diverse modelling techniques. For example, typical models for cardiac mechanics simulation (e.g. [16, 31, 32, 40]) fundamentally use a FEM approach, but require additional structures that are not common in traditional FEM applications. One case presents a left ventricle model using finite element interpolation of a geometry field to represent the anatomy, and a rotation field to represent the cardiac myocyte fibre and laminar fibre sheet orientation [25], with care taken to ensure that only small-angle rotations are modelled, since FEM interpolation is only able to approximate the geodesic in rotation space when directly interpolating Euler angles [37]. In another case, diffusion tensor magnetic resonance (DTMR) data are modelled using FEM, and a computationally efficient compromise is preferred between fast but inexact tensor Euclidean interpolation and exact tensor interpolation [15, 16]. Neither of these features is typical for standard FEM interpolation, and, to our knowledge, neither is supported in any existing mainstream open FEM data formats. For models that couple cardiac electrophysiology with cardiac mechanics, not only is it necessary to represent the geometry of the organ and other anatomical tissue structures, but it is also necessary to represent the cellular electromechanical model. CellML is already a well-established cellular model representation system, well suited to representing the ordinary differential equation and differential algebraic equation (DAE) models typical for cardiac electrophysiology. It is also used to represent algebraic material constitutive laws for mechanical modelling, as well as a wide range of other modelling areas, extending even beyond physiological modelling [28]. OpenCMISS [3] and Chaste [33] are simulation systems that support a type of multiscale modelling by the coupling of FEM models with CellML cellular models. In these systems, a standard FEM field can be used to represent the values of any parameters of the CellML model that may vary spatially. Also, in some cases, different cellular models are used in different spatial regions. A future goal of FieldML is that it will be able to represent these links, and this has influenced the current design, as is described later in this article.

Representing the link between clinical images and the resulting patient-specific models by means of image annotations [1, 12] or fitting models and model fields to image data [16, 40] is also a common requirement. If imaging or other clinical data are used to identify diseased spatial regions, then fields can be used to represent the relevant spatially varying cell model parameters. Traditional FEM approaches tend to follow a workflow from computer-aided design (CAD) software to FEM model and hence tend not to support linking to medical imaging and other clinical data directly. As is described later in this article, FieldML supports field definitions that relate data in multiple data sources of different kinds, and hence can be used for representing these links.

Although existing software and formats were often sufficient for the modelling work done in euHeart, a key goal of euHeart was to enable software and models to be free from licensing restrictions. CellML, FieldML and PMR are being developed as open standards and open source software, and the Physiome Model Repository site (http://models.physiomeproject.org) uses the PMR software to house numerous models, the vast majority of which are licensed under a Creative Commons licence.

BioModels [27] and the Anatomical Model Database (AMDB) [14, 20] are similar model databases, BioModels focussing on SBML [17]. Embracing this linked open science approach [19] has the significant benefit of lowering the barrier to collaboration, making data, models and tools easily available. These approaches are essential to meet the challenges that face projects similar to euHeart, and the wider Physiome project, and many other similar fields [18]. Hence, the FieldML design work has benefitted from being part of the euHeart project and makes it possible for other euHeart work to be available for follow-on projects.

## FieldML and related technologies

In the following subsections, FieldML is compared to similar formats, and we show how the FieldML design is related to CellML, and how the PMR software supports FieldML.

### Brief comparison with formats with similar goals to FieldML

There are numerous file formats that are used with FEM computational and visualisation software (see e.g. the lists at the ParaView FAQ^3^ and the VisIt FAQ^4^). In [7], some relevant formats and software libraries were discussed, for example GENERAL MESH VIEWER format,^5^ EXODUS II format [36], Sets and Fields (SAF) modelling system [30] and libMesh [21]. As discussed in [7], these formats do not meet the goals of FieldML because they are not general enough for the requirements of Physiome modelling. As the design and development of FieldML has continued since [7] was published, some new design approaches have emerged in the FieldML work. Since discussion of all open FEM formats is beyond the scope of this article, we chose to discuss only formats that appeared to us to have some overlaps with these new aspects and were not previously discussed.

The eXtensible Data Model and Format (XDMF) and FieldML version 0.5 share the design approach of segregating heavy data from light data and also the use of XML to describe light data [8]. The term *heavy data* refers to the data that consists primarily of large arrays of homogenous data, usually numerical data, used, for example, for the values of a field at the mesh nodes. *Light data* refers to the data that describes the meaning of the heavy data and is usually smaller in size relative to the heavy data, for example, stating the total number of mesh elements and nodes, and the mapping of the heavy data relative to the field interpolation method used for a mesh. XDMF stores heavy data using HDF5.^6^ In FieldML version 0.5, using HDF5 is one of the options for storing heavy data. Also, the FieldML API version 0.5, like the XDMF API, is implemented in C ++ and wrapped such that it can also be used from popular languages (Java and Fortran in the case of FieldML version 0.5; Java, Python and Tcl in the case of XDMF).

CGNS [24, 39] abbreviates “CFD General Notation System” and is an open standard with supporting open source software. The CGNS design does not have any inherent limitation that prevents its use outside of computational fluid dynamics (CFD).^7^ This extensibility is also a goal of the FieldML project, both for existing version and as future versions are designed. As indicated by the format name, the current standard CGNS labels are primarily focussed on the CFD subject area, and usually in an aerodynamics context [24]. CGNS appears to have good adoption and support [24]. CGNS serialisation uses HDF5 for both heavy and light data. As an alternative to HDF5, a legacy custom format called the Advanced Data Format (ADF)^8^ can also be used.

The Visualization Toolkit (VTK) provides its own file format,^9^ which uses either a plain text file following the VTK syntax or an XML file. In either case, both the heavy data and the light data are stored in the same file. VTK appears to have wide adoption in Physiome research.

VTK, CGNS and XDMF all rely on standardised strings, and this is common practice for many of the formats in this field. This is used, for example, for describing FEM element shapes,^10^,^11^ or geometric coordinate systems^12^ [8], and hence, extension would rely on users agreeing on conventions for new standard string labels. As discussed in [7], it is advantageous if these aspects can be described in FieldML itself in such a way that extension is usually possible without relying on new conventions. A current feature of FieldML version 0.5 is that this is done by using “external evaluators”, described in Sect. 5.1, a syntax that is forward-compatible with the planned future syntax for describing element shapes and the algebraic form of interpolation functions. However, like VTK, CGNS and XDMF, it currently relies on current software adhering to conventions for the meaning of the string names used for the external evaluators.

### Physiome Model Repository software support for FieldML

The PMR software supports a plug-in architecture, which allows plug-ins to be added to PMR in order to support different ways of viewing the models in PMR. The first versions of the PMR software focussed on the support of CellML representations of published models. The model repository also contains numerous CellML representations of cardiac models, including cardiac circulation models, tissue mechanical constitutive laws, excitation–contraction coupling models and cardiac electrophysiology models [28]. Many of these are used for euHeart simulations, for example, the CellML representation of the ten Tusscher–Panfilov model^13^ [5, 38]. The PMR site provides a robust point of access for these models, allowing euHeart researchers a reliable site for retrieving models that they require for modelling, and for depositing models produced by their research.

More recently, in order to demonstrate how FieldML can be supported, a PMR software plug-in was developed and deployed at http://models.physiomeproject.org/fieldml, which supports visualisation of exnode/exelem models [41], and has been recently adapted to allow for visualisation of FieldML version 0.5 models. This plug-in allows the models to be viewed via an interactive 3D view, by means of the Zinc web-browser plug-in.

**Fig. 1:**
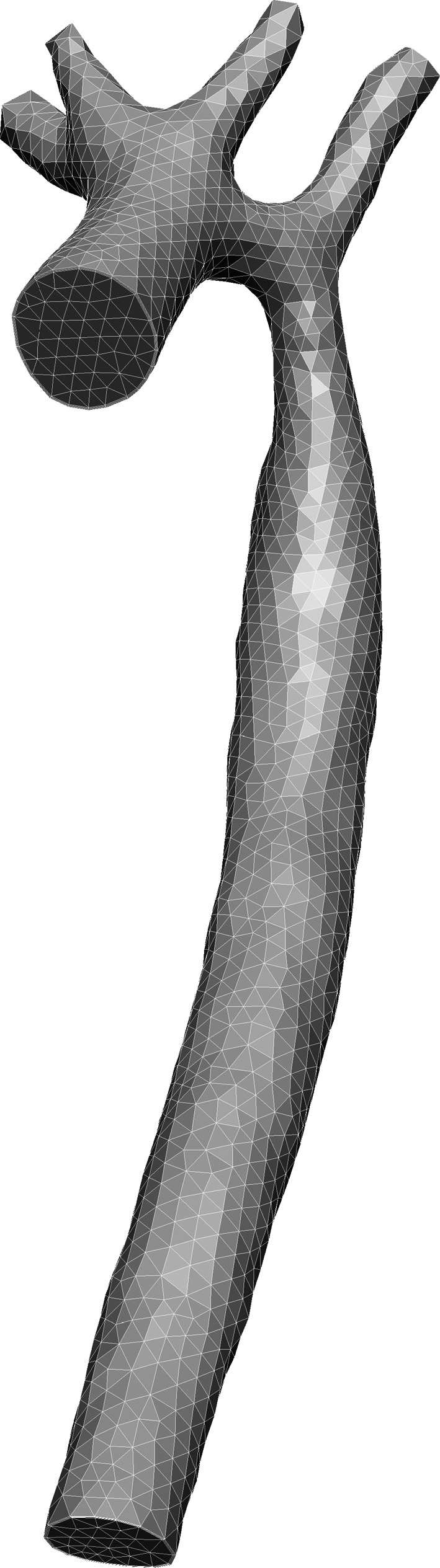
An euHeart aorta model. This is an aortic coarctation model with four vessel branches in the aortic arch: the right subclavian artery, right common carotid artery, left common carotid artery and the left subclavian artery. The figure shows a coarse mesh for demonstration purposes. In the CFD calculation, a much denser mesh was used, with 375695 nodes, 555027 tetrahedral elements and 543720 prism elements, using a different format [4]

**Fig. 2:**
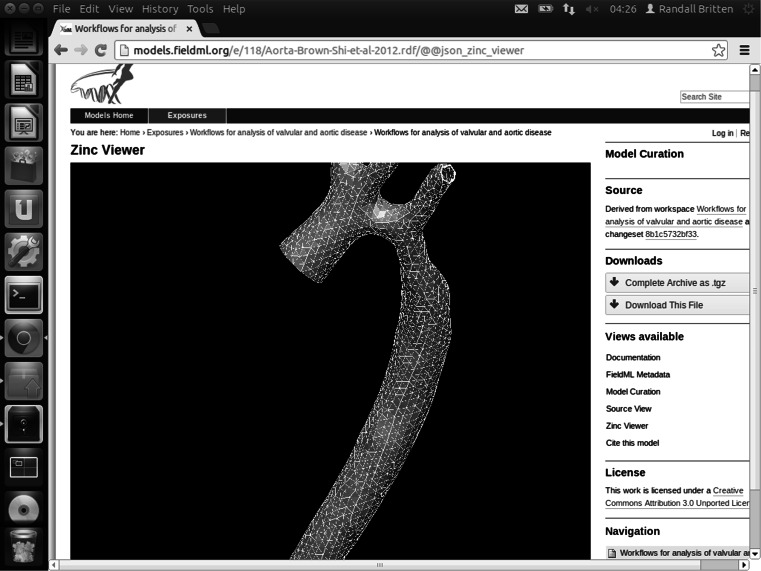
Screenshot of an interactive 3D visualisation of the FieldML version 0.5 representation of the aorta model, embedded in a web page served by the PMR software

There are currently only six models in the FieldML portion of the repository. One such model is shown in Figs. 1 and 2 and is available via the model repository, at http://models.fieldml.org/e/118/Aorta-Brown-Shi-etal-2012.rdf/view. The PMR site provides hosting for this data, making it publically accessible. The PMR software also provides version control and provenance services [41]. Version control of groups of related files is a key feature of the PMR software that was originally developed to support CellML model imports (see [41] for details). Because FieldML version 0.5 files can refer to other FieldML files, or to external data located in different files,^14^ the version control feature of the PMR software has made it ideally suited to house FieldML models that are made up of groups of related files, as is the case in the above example.

**Fig. 3:**
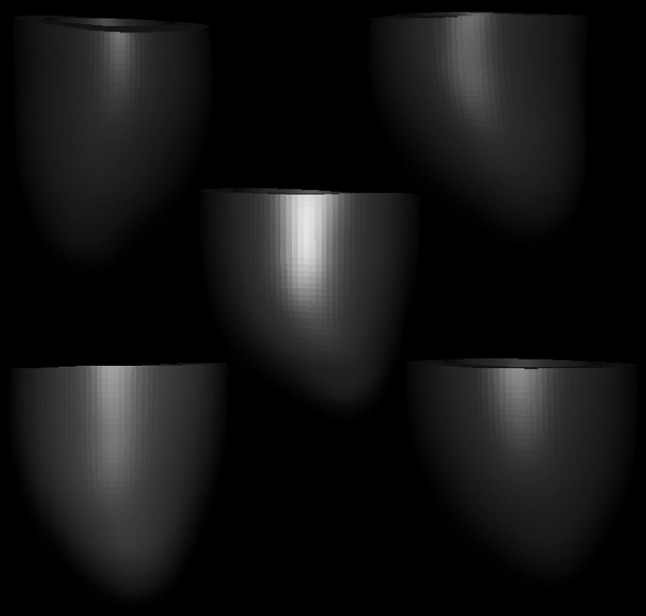
Visualisation of FieldML representation of five patient-specific human left ventricle anatomical models

The AMDB also hosts a number of models in exnode/exelem format for euHeart, with the associated 3D interactive Zinc viewer. Unlike PMR, AMDB focusses on anatomical models, whereas PMR initially focussed on CellML models, and still only has a small number of anatomical models compared to the AMDB. One group of models that it contains consists of anatomical data for the human left ventricle that was constructed from a cohort of young subjects [26] using the methods described in [22], as part of the euHeart project. A subset of these was selected as part of the demonstration work for FieldML, and a FieldML version 0.5 representation is available in the AMDB^15^ (see Fig. 3). This example also makes use of FieldML version 0.5’s support for HDF5, which facilitated capturing anatomy for multiple subjects within one data source.^16^ In the previous versions of FieldML, this would have required separate data source files for each subject’s anatomical data.

### Comparison of CellML and FieldML

CellML and FieldML have a number of similarities and differences. As already mentioned, CellML focusses on lumped parameter modelling. FieldML’s focus is modelling spatial–temporal variation and, more generally, multidimensional differential and topological manifolds. Both rely on XML as a serialisation format, and in this regard, CellML is relatively mature and has broader adoption than FieldML. CellML relies heavily on MathML [6] to represent algebraic and differential equations. Reliance on MathML or OpenMath (see the OpenMath standard documentation^17^) has always been envisaged for FieldML, and the current design is intended to be forward-compatible with a planned future extension to support this. Nevertheless, this is still an area of active research, and released versions of FieldML have not yet incorporated MathML support.

Serialisation of CellML and FieldML entails conversion of in-memory software data structures to a persistent form, usually stored in a computer file system, but also necessary in other scenarios, for example, when transmitting data over a network. Deserialisation is the conversion in the opposite direction, for example, reading data from the file system and creating the original data structures in the computer’s memory for the software to process and manipulate^18^ [11].

The CellML API is discussed in detail in [29], which highlights the benefits of having an API to accompany a standard format. It has features to support serialisation and deserialisation, as well as facilities for commonly needed processing of CellML models, such as model validation and simulation. Providing an API to accompany a standard format also helps ensure that different software applications that work with the format will interpret the format in a manner consistent with each other if they rely on the API for much of that interpretation. In the hope that similar benefits will be achieved for FieldML, a similar API has been developed to accompany FieldML version 0.5, and this is discussed in the next section.

There are other minor aspects of the CellML design that have influenced the FieldML design and implementation, for example, the use of the simple linking subset of the XML Linking Language, Xlink [10], as the mechanism by which one FieldML file can make reference to another FieldML file, or the use of the libxml2 library^19^ for low-level parsing of XML by the FieldML API, as has been done for the CellML API. As mentioned previously, PMR features to support CellML have been able to be adapted to address similar issues required to support FieldML.

## The API for FieldML version 0.5

The design work on the FieldML version 0.5 API has focussed on serialisation and deserialisation. Serialisation allows the in-memory representation of an explicit model to be transferred from a software program to a file on the computer system’s persistent storage according to the FieldML file format. Deserialisation allows software to recover an in-memory representation of the explicit model from persistent storage. This obviously allows for explicit models to be transferred between two different applications via the persistent storage. It is often desirable to transfer the model representation directly between two different software applications without going via the persistent storage. However, an API for communicating information about fields directly between applications is not yet part of the FieldML version 0.5 API. This idea is, however, common to representation formats; see, for example, the OpenMath standard documentation.

**Fig. 4:**
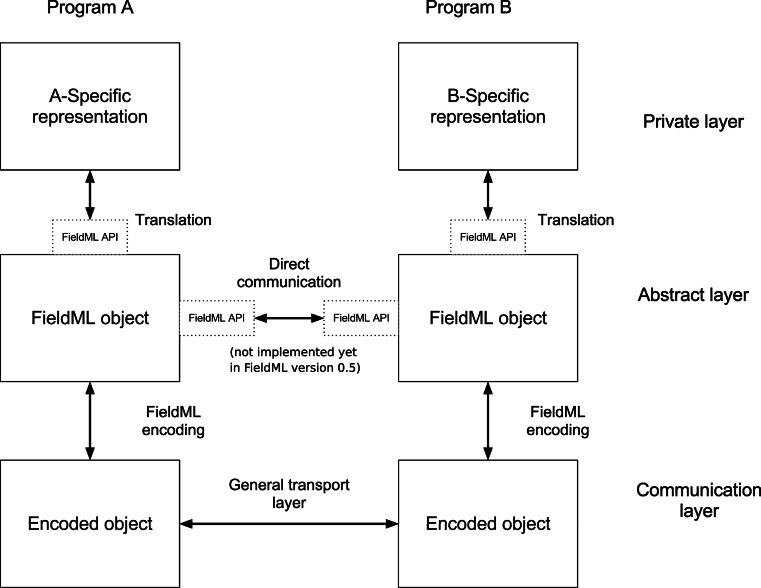
FieldML serialisation and communication architecture

To illustrate this, Fig. 4 shows a schematic view of the field representation layers in two different hypothetical applications, and how the FieldML format and the FieldML API could be used to exchange field representation objects. This diagram is analogous to the OpenMath architecture diagram (see the OpenMath standard documentation), but includes not only the serialisation format, but also the use of an API. The private layer of each application is its own internal field representation. The abstract layer represents fields according to the FieldML object model. In the communication layer, fields are represented by an XML encoding of the FieldML objects, or a mixture of formats (e.g. XML and HDF5). It is also possible for applications to directly use the FieldML object model as their private representation, or to omit the FieldML object model representation altogether, directly translating from their own internal representation to the FieldML serialisation format. For the purposes of validating the API design, we implemented a demonstration that allows for limited FieldML version 0.5 exchange between cmgui^20^ and OpenCMISS [3], two existing applications. Note that the API implementation does not yet have any features for calculating the numerical values of fields, and each application is still required to have its own implementation for the evaluation of fields.

## FieldML serialisation design progress

The general FieldML conceptual design has existed for some time [7]. Our focus over recent years has been on designing an XML serialisation for FieldML, consistent with these general ideas, as well as improving the conceptual design itself. An older format used by the CMISS software system since the 1990s, although not based on XML, has had a very direct influence on the initial FieldML design. It consists of “exnode” and “exelem” text files and is also called the “ex-format”. However, it was never intended as a standard for model interchange. FieldML version 0.1, the first XML version, was developed in 2005 and is supported by Cmgui and CMISS. However, no special-purpose API was created for version 0.1, and it followed the same overall structure as the ex-format.

In 2010, FieldML version 0.2 was released as an XML specification described by an XML Schema document (XSD), along with the first FieldML API, which focussed on supporting serialisation and deserialisation.

Rather than just being an incremental update of version FieldML 0.1, FieldML 0.2 started afresh, aiming to create a design, which could be incrementally developed towards the goal of a general mathematical model representation format. FieldML version 0.5 is the most recent version and was released in May 2012. It supports representation of models that use rectangular Cartesian coordinates and isotropic interpolation. The overall goal of FieldML is relatively ambitious, and it was necessary to prioritise the planned features and incrementally introduce these. Thus, versions from FieldML 0.2 up to and including FieldML 0.5 are limited to explicit field description, meaning that the field descriptions are equivalent to explicit algebraic expressions. This contrasts with CellML, where the model describes the solution functions implicitly, by means of series of Boolean predicates, which are asserted to hold true, and which might, for example, specify an ODE system or a DAE system. Thus, FieldML version 0.5 cannot represent implicit fields and thus cannot represent partial differential equation systems.

Table 1 shows a summary of the features introduced in each version of FieldML since FieldML 0.2, and a comparison with the precursor formats.

**Table 1:** Feature comparison between versions of FieldML and precursor format

Feature	Exnode Exelem	FieldML				
		v0.1	v0.2	v0.3	v0.4	v0.5
Technical						
XML		✓	✓	✓	✓	✓
Independent FieldML API			✓	✓	✓	✓
External data sources					✓	✓
Imports					✓	✓
HDF5 support						✓
Parallel I/O^a^						✓
Model						
1D elements	✓	✓	✓	✓	✓	✓
2D Quadrilateral elements	✓	✓	✓	✓	✓	✓
3D Hexahedral elements	✓	✓	✓	✓	✓	✓
2D Simplex elements	✓	✓		✓	✓	✓
3D Simplex elements	✓	✓			✓	✓
Boolean evaluators						✓
User-defined tensor products of bases	✓	✓				
Curvilinear coordinates	✓	✓				

^a^Parallel I/O is made possible due to the use of HDF5. However, only very basic testing of this feature has been done

## The FieldML version 0.5 data model

This section gives a brief overview of the FieldML 0.5 data model. The FieldML 0.5 file format makes use of XML to represent data according to this model, and details of the XML file format are provided in Appendix A (ESM). Section 6 gives an illustrative example. While these ideas are essentially the same as those described in [7], some advances have been made as the design work has progressed.

Conceptually, a FieldML version 0.5 model consists of descriptions of the FieldML domains and the fields of the model. FieldML domains are essentially just mathematical sets with some additional structures that convey topological and possibly other information. Fields are essentially just mathematical functions, where the definition of the function leverages the information about the FieldML domains. As is standard in mathematics, the function domain^21^ and codomain of the function are specified, either directly or implicitly. The standard mathematical shorthand for describing a function’s domain and codomain is the function, a colon, the domain, a right arrow and the codomain, for example, for a function *f* with domain *A* and codomain *B*, this is written:

$$f:A \to B$$

Compatible fields may be composed together, in a manner similar to mathematical function composition.

Currently, FieldML relies on its specification to define certain FieldML domains and fields that cannot yet be described by the FieldML language itself, such as a collection of interpolation functions, and common FEM element shapes. (Note: the term “chart” is sometimes preferred to “element”, due to the influence of smooth manifold theory [23] on the design of FieldML. “Chart” avoids confusion with “XML element” in the context of the XML representation of FieldML 0.5, and also “element of a set” in the context of set theory.) Nevertheless, these functions and element shapes are listed in the FieldML library, which is just an ordinary FieldML file, and are declared there as if they were defined elsewhere.^22^ This is because we plan to introduce the facility to represent their descriptions in FieldML itself. Recent design work^23^ on a future version of FieldML includes design proposals for how to represent these descriptions. Nevertheless, current software needs to recognise these objects by their string identifiers, and they may be used by reference in FieldML documents.

In FieldML 0.5, fields are defined through the composition^24^ of a series of compatible evaluators to form an evaluator pipeline, similar to the composition of mathematical functions. A field in version 0.5 is therefore synonymous with an evaluator pipeline. The number of possible ways of defining fields by means of different evaluator pipeline compositions is essentially limitless, and this innovative approach gives FieldML version 0.5 broad expressive power.

FieldML version 0.5 does not yet fully support the original vision for the range of ways that different domains can be represented, as described in [7]; rather it relies on *types*; nevertheless, in the descriptions that follow, the terms domain and type are used interchangeably.

More details on how evaluators and their pipelines, and types are defined are provided in the following subsections. The detailed XML syntax is described in Section 11 which is in Appendix A (ESM), and the relevant references to the subsections of Appendix A (ESM) are given where appropriate.

### Evaluators

There are seven ways to define an evaluator: argument, parameter, piecewise, aggregate, reference, external and constant, each of which will be discussed in turn.

Fields are defined over domains and may, in general, vary between points in the domain. FieldML version 0.5 can deal with this variation in fields over a domain by treating an evaluator pipeline as a function, with the point in the domain on which the field is defined as an input to (domain of) the function, and the value of the field at that point as the output (codomain) of the function.

An *argument evaluator* allows references to be made to this functional domain. Individual argument evaluators name a specific argument. An argument is more than just a value from a domain, such as the set of real numbers; it also attaches semantic meaning (e.g. “time” could be an argument). Different argument evaluators in the same evaluation pipeline may refer to different arguments; in this case, each argument evaluator only refers to a component, or factor, of the domain over which the field is defined.

Consider, for example, an evaluation pipeline that contains only one evaluator, an argument evaluator. The field will be equivalent to the identity function over the argument. An evaluation pipeline that takes two argument evaluators, for real-valued arguments *x* and *y*, respectively, and adds^25^ them would be equivalent to a function from ${\mathbb{R}}^{2}$ to ${\mathbb{R}}$, where ${\mathbb{R}}$ is the set of real numbers.

(The specifics of using XML to define an argument evaluator are provided in Sect. 11.7).

A *parameter evaluator* describes piecewise functions from *N* discrete, finite-membered discrete domains (called ensembles and discussed later) to some other space (which must be either a scalar continuous type or an ensemble type), by looking up values in stored data. Individual parameter evaluators refer to one or more delegate evaluator inputs, called indices, and a reference to data that describes the output values corresponding to each input value. The data array can be dense over any index (meaning that for all values of the index in a contiguous range, there is a corresponding parameter value) or sparse over any index (meaning that a parameter value exists only for some of the index values in a contiguous range). For example, a parameter evaluator could define a mapping from a node identifier to a point in the 2D Cartesian plane represented as the tuple *(x,y)*, where *x* and *y* are the Cartesian coordinates:

$$f(n) = \left\{ {\begin{array}{*{20}c} {(0,0)\quad if\; n = 1} \\ {(1,0)\quad if\; n = 2} \\ {(0,1)\quad if\; n = 3} \\ {(1,1)\quad if\; n = 4} \\ \end{array} } \right.$$

(The specifics of using XML to define a parameter evaluator are provided in Sect. 11.8).

A *piecewise evaluator* defines a piecewise function from a discrete domain to the codomain. Usually, piecewise evaluators serve as the final step in the pipeline for defining a field over a FEM mesh. The discrete domain (called the index) usually identifies an element in the mesh. The codomain is defined by input-dependent references to evaluators (called delegate evaluators). The function domain of each of the delegate evaluators is a domain that represents the element shape. For example, the value of a field over a mesh may use a different type of interpolation within different elements. An example of a piecewise function that can be represented by a piecewise evaluator is as follows. For the delegate evaluators *g* and *h*, an evaluator *f* can be defined using a piecewise evaluator:

$$f(n,\xi ) = \left\{ {\begin{array}{*{20}c} {g(\xi )\quad if\; n = 1} \\ {h(\xi )\quad if\; n = 2} \\ \end{array} } \right.$$

Here ξ is the parameter for the location within the relevant mesh element.

(The specifics of using XML to define a piecewise evaluator are provided in Sect. 11.9).

An *aggregate evaluator* is structurally similar to a piecewise evaluator, except that it is used to define a vector^26^ value by defining each component of the vector. In an aggregate evaluator, each item of the vector is identified by an index from a specified ensemble (with data ordered in the numerical order of member identifiers in that ensemble). For example, this would allow for a constant three-dimensional Cartesian vector *(0.5,*−*1, 20.1)* to be defined.

(The specifics of using XML to define an aggregate evaluator are provided in Sect. 11.10).

A *reference evaluator* allows an evaluator to be created through reference to another evaluator. Used by itself, this creates an evaluator that is an alias for another evaluator. However, reference evaluators are most useful when used to bind argument evaluators (discussed below).

For example, if an existing evaluator, *f(x)*, had already been defined, a new evaluator *g(x)* could be defined by reference, essentially equivalent to stating that *g* = *f*.

(The specifics of using XML to define a reference evaluator are provided in Sect. 11.11).

*External evaluators* declare additional evaluators, providing an extension mechanism. The semantics represented by an external evaluator must be described by a convention, usually just by means of an accompanying (not necessarily machine readable) document. These external evaluator types are generally referenced by a reference evaluator, which makes use of the binding functionality to associate the arguments with the external evaluator with user-defined delegate evaluators. External evaluator types are used for many different types of function in FieldML documents (e.g. to define fields as an interpolation from other fields). A standard library of external evaluator types is presented in Sects. 5.7 and 11.16.

(The specifics of using XML to define an external evaluator are provided in Sect. 11.11).

Finally, *constant evaluators* represent constants, for example, the real number 1059.87 or the integer 57. FieldML version 0.5 has the limitation that there must be a supported way of representing the constant value as a string, and so constant evaluators on their own cannot be directly used to represent data that has more complexity, such as a constant vector or a constant matrix. However, in combination with other evaluators, such as aggregate evaluator, constant objects with more complexity can be represented.

(The specifics of using XML to define an external evaluator are provided in Sect. 11.11).

### Binding

The reference evaluator, the piecewise evaluator and the aggregate evaluator all allow bindings to be defined. Bindings associate an argument evaluator input into an evaluation pipeline with another delegate evaluator. It is essentially equivalent to substituting terms of mathematical expressions. The substitutions mean that the current evaluator connects the delegate evaluator to a connection point that is upstream in the evaluation pipeline. The connection point is always an argument evaluator that is referred to by the definition of an evaluator upstream of the current evaluator. The binding makes this connection by specifying the name of that argument evaluator.

For example, a piecewise evaluator may be defined as follows:

$$g(n) = \left\{ {\begin{array}{*{20}c} {7.1\quad if\;  n = 1} \\ \begin{gathered} 100\quad if\; n = 2 \\ x\quad if\; n = 3 \\ \end{gathered} \\ \end{array} } \right.$$

Here,* x* stands for an argument evaluator upstream of *g*.

A reference evaluator could then reference *g* and use binding to bind *x* to a constant evaluator. In other words, a constant is substituted for *x*. So, using the equation *k*1 = 0.331 to represent a constant evaluator, binding would be equivalent to defining a new function:

$$f(n) = g(n)\;{\text{such}}\,{\text{that}}\;x = k1.$$

In the above example, the argument evaluator *x* is a scalar. Argument evaluators can also represent functions, and then binding specifies that the bound evaluator be applied to the operands of the function. For example, if instead we had

$$g(n) = \left\{ \begin{array}{ll} 7.1& \quad \hbox{if } n = 1 \\ 100& \quad \hbox{if } n = 2 \\ h(29)& \quad \hbox{if } n = 3 \end{array} \right.,$$

then binding was done as follows:

$$f(n) = g(n)\;{\text{such}}\,{\text{that}}\;h = s,$$

which is equivalent to using *s(29)* for the case *n* = *3*.

As discussed above, bindings are commonly used with reference evaluators referring to external evaluators. For example, a user might use the bilinear Lagrange interpolator external evaluator (defined in a library as discussed later under the section “Imports”) and bind evaluators in their FieldML description to the interpolation parameters, to give a new evaluation pipeline describing an interpolated field.

A more complex example that uses binding is provided in Sect. 6, with the details supplied in the supplementary material.

(The specifics of using XML to define bindings are provided in Sect. 11.6).

### Domain types

In FieldML 0.5, *domain types* are defined as possibly infinite sets of values. Domain types may be continuous, or discrete. Every evaluator in a model must have a *value type*; the value type references a domain type defined in the model.

FieldML 0.5 has four kinds of domain type: ensemble types, Boolean types, continuous types and mesh types.

*Ensemble types* are discrete types for describing countable sets of objects or entities. The allowable values for an ensemble type are called members and are declared as part of the ensemble definition. For example, an ensemble can represent the set *A* = *{4, 37, 60, 1002}*.

In FieldML 0.5, members of an ensemble are defined with unique non-negative integer identifiers. Like mathematical sets, ensembles are conceptually unordered. However, as discussed later, an order is imposed for the purposes of serialising data indexed by the ensemble. Applications may discard the ordering or the member identifiers on an ensemble type (or both) once they are no longer required to interpret the model.

(The specifics of using XML to define ensemble types are provided in Sect. 11.2).

*Boolean types* declare the canonical Boolean type, i.e. the discrete set with two elements: True and False. FieldML v0.5 does not have any support for Boolean operators such as “and”, “or” and “not”, so Boolean types serve only as the field type for the predicates that are used to define element shapes (see below).

(The specifics of using XML to define Boolean types are provided in Sect. 11.3).

*Continuous types* describe continuous *n*-dimensional domains. For dimension exceeding one, FieldML 0.5 implicitly defines a corresponding ensemble for indexing the components of the vector representing a value of the *n*-dimensional domain. For example, a continuous type can be used to declare the set of all points in ${\mathbb{R}}^{3}$.

(The specifics of using XML to define continuous types are provided in Sect. 11.4).

Roughly speaking, *mesh types* essentially describe a FEM mesh. In FieldML 0.5, all elements of a mesh are of the same dimension, and this is specified as part of the mesh definition. Each element is itself a continuous domain. In FieldML 0.5, the FEM element shape definitions rely on an external definition. Nevertheless, they are declared in the FieldML library by means of Boolean-valued external evaluators. The evaluator defining the field is called the predicate. The predicate yields the value true within and on the boundaries of the shape and false outside those boundaries. As mentioned earlier, current software processing FieldML 0.5 will recognise the string names in order to process the shapes, but this design is forward-compatible with a proposal for a future version of FieldML where algebraic expressions will be used to define the predicates. It is not expected that processing software will blindly attempt to discover the shape by evaluating the predicate at numerous points, but rather that it will inspect the definition of the predicate itself, which allows for direct processing of the represented shape.

Each mesh type definition also implicitly defines an ensemble type and a continuous type. Each mesh type definition also implicitly defines a mechanism to access the element and component. The definition of the mesh itself does not imply anything about the connectivity of the elements.

See Sect. 6 for an example of a mesh.

(The specifics of using XML to define ensemble types are provided in Sect. 11.5).

### Strong typing

The term “domain type” reflects a conceptual dichotomy where, on the one hand, a domain type represents a mathematical set, often with a spatial interpretation, and, on the other hand, a domain type represents a data type akin to data types in common programming languages such as C++ and Java. Both views are valid ways of thinking about domain types. Taking the data type view, FieldML 0.5 is strongly typed. For example, members of an ensemble *B* = *{1, 2, 3}* are incompatible with members from any other ensemble type, for example, *C* = *{1, 2, 3}*, even though they have the same identifiers. Incompatibility means, amongst other things, that function composition would not be valid. For example, if $f:A \to B$ and $g:C \to D$, then one cannot perform function composition $g \circ f$. To reference the values of one ensemble given another ensemble with the same identifiers, a field that maps from one ensemble to the other needs to be defined, so for the above example, we could define

$$h:B \to C$$

such that

*h(1)* = *1*

*h(2)* = *2*

*h(3)* = *3*

**Fig. 5:**
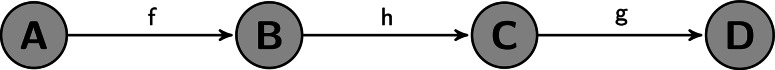
Using an intermediate conversion function to allow for compatible composition of evaluators

This allows the composition $g \circ h \circ f$ to be formed (Fig. 5).

### Data resources and data sources

A FieldML 0.5 *data resource* is a link to raw/bulk data serialised as an in-line string, external text file or HDF5 data set. A data resource declares one or more *data sources* which each mark up a part of the resource as a dense array of zero or more dimensions. This is another innovative design aspect that permits many existing data files to be incorporated into FieldML as one data resource, with separate sections, rows, columns or subarrays marked up as distinct data sources. Data sources play an important role in defining FieldML models, for example, serving to provide the values for the degrees of freedom (DOFs) of interpolated fields, and providing element local node to mesh global node mappings. When the data sources are used to describe external data, it is often possible to use file positions in existing data formats. This means that other data formats can be “wrapped” by FieldML and minimises the need for data conversion.

For example, if the data representing the mapping from the four local nodes of each square element of a FEM mesh made up of square elements was represented as a matrix with four columns (one column for each local node) and one row for each mesh element, with entries representing global node number, the data source would essentially contain data that looked something like the following:

**Table:** 

1 2 11 12 2 3 12 13 3 4 13 14 … 89 90 99 100	

(The specifics of using XML to define data resources and sources are provided in Sect. 11.14).

### Imports

In software engineering, there are a number of widely recognised techniques for managing software complexity, some of which are also relevant to the representation of field models.

One such property is abstraction: to understand one part of a system, it is only necessary to understand the abstract properties presented by another part. Another such property is modularisation; a system is described through the composition of different modules.

To allow FieldML models to be developed using abstraction and modularisation, evaluators and types that are defined in one FieldML file may be imported into another. This mechanism has essentially been borrowed from the CellML import feature (see [9]), but is considered innovative with regard to field formats and FEM formats. This allows for modularity, because libraries of functions can be defined in one file (a module) and composed in the file that imports them. Abstraction is possible because the imported evaluator may be defined as a pipeline of other evaluators, which are not themselves imported, but which provide the details that are abstracted away.

The best example of this is when a model imports the evaluators and types declared in the standard library for FieldML version 0.5 (described next). Such an example model is presented in Sect. 6.

### The FieldML 0.5 standard library

Because the FieldML 0.5 evaluation model is very generic, most of the specific functions needed to build useful evaluation pipelines need to be defined using the external evaluator functionality.

External evaluators are meaningless without a specification of how they are evaluated. In addition, FieldML models can only be meaningfully exchanged between tools that support all external evaluators used in the model. Therefore, it is important that some of the more fundamental external evaluators have a standardised definition.

The FieldML 0.5 standard library defines (using the FieldML language itself) a set of external evaluators, along with the types required by these standard external evaluators, and argument evaluators for with those types. This standard library is available at http://www.fieldml.org/resources/xml/0.5/FieldML_Library_0.5.xml.

In summary, the library specifies:

- 1D, 2D and 3D Cartesian coordinates as continuous domains
- Ensembles for indexing Cartesian coordinate domains
- 1D, 2D and 3D coordinates for elements of corresponding dimension
- Declarations of external evaluators for common FEM interpolation basis functions
- Ensembles for indexing these basis functions (for nodal value interpolation, these are essentially the element local node indexes).
- A range of element shapes (via Boolean external evaluators).
- Argument evaluators for the above ensembles, continuous domains and the ensembles that index the components of multidimensional continuous domains

The contents of the library and the meaning of its external evaluators are described in detail in Appendix A (ESM) (see Sect. 6).

## FieldML 0.5 illustrative example

**Fig. 6:**
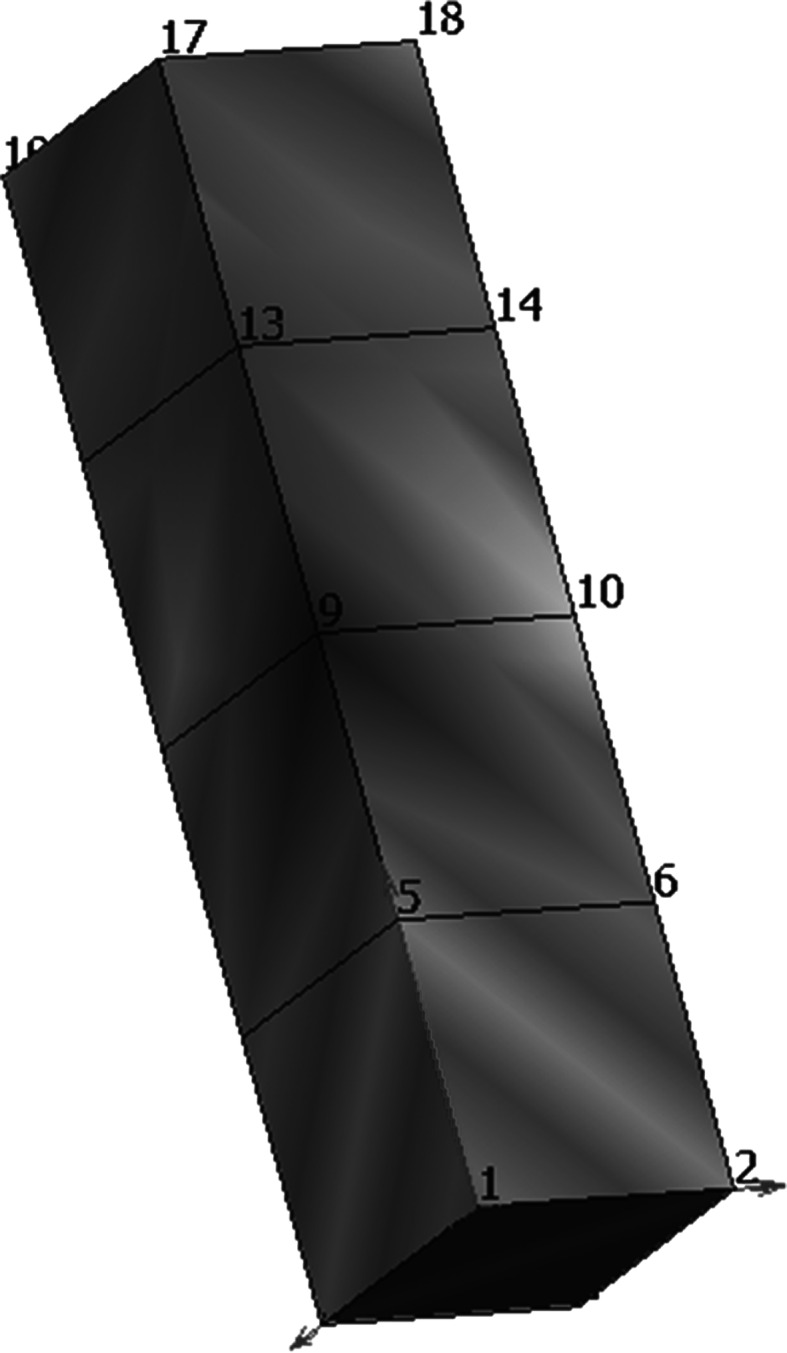
Simple FieldML 0.5 example, colour variation represents the scalar pressure field

This section gives an overview of a simple FieldML 0.5 example of a FEM mesh, for the purpose of illustrating how the FieldML building blocks described in Sect. 5 are assembled to create a model. The full listing of the FieldML XML is provided as supplementary material and accompanied by a line-by-line explanation that also provides mathematical notation of the domain and field definitions of the example. FieldML is used to represent the geometry of the mesh, as well as a scalar field (which represents some measure of pressure in this example). A visualisation created using cmgui is shown in Fig. 6.

**Fig. 7:**
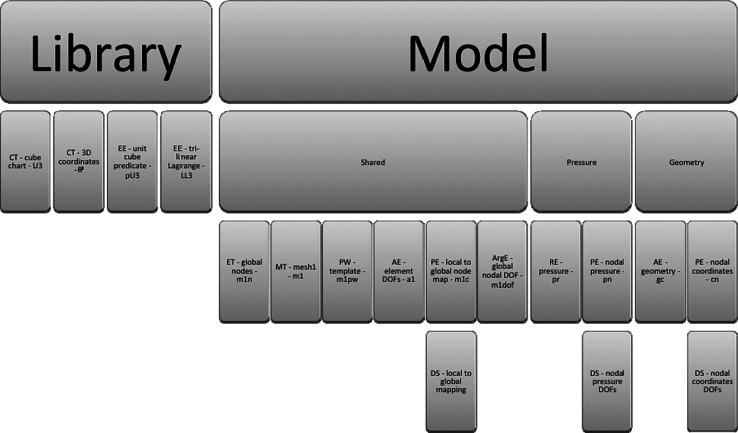
Schematic overview of the example FieldML model XML and mathematical structure, the figures that follow after this show detail on parts of this schematic. Key: *CT* continuous type, *EE* external evaluator, *ET* ensemble type, *MT* mesh type, *PW* piecewise evaluator, *AE* aggregate evaluator, *PE* parameter evaluator, *DS* data source, *ArgE* argument evaluator, *RE* reference evaluator. Structure is shown for the relevant parts of the standard FieldML library, and for the example model. Each evaluator or type entity’s label consists of the abbreviation of the type according to the legend, a brief description and the symbol used in the mathematical representation describing the example (see line-by-line annotation in supplementary material). See the following figures for details on each of the major sections of this schematic

**Fig. 8:**
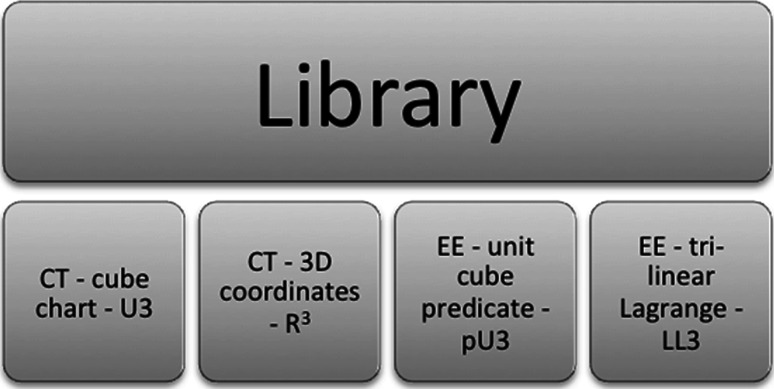
Detail of imports from library used in example model (see overview schematic for the explanation of abbreviations)

**Fig. 9:**
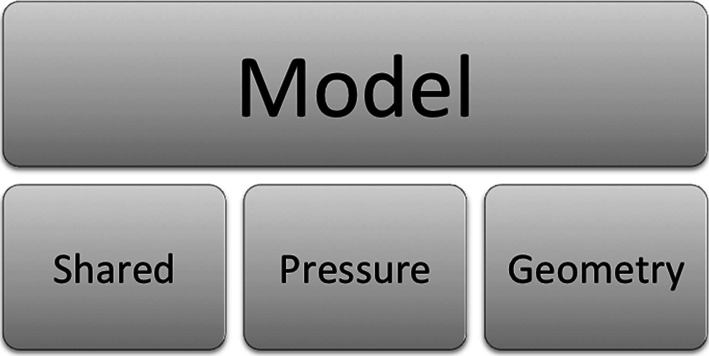
More detail of model structure. The structures in the example model are grouped by relevance to the pressure field, the geometric field or shared structures, and detailed schematics for these are presented in the figures that follow

**Fig. 10:**
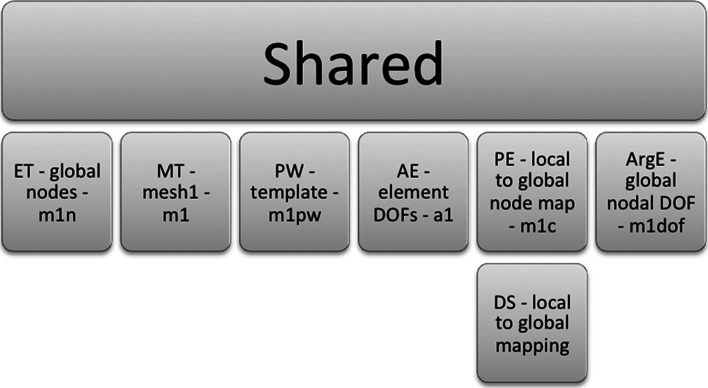
Detailed schematic for the part of the model shared by both the field definitions for pressure and geometry (see overview schematic for the explanation of abbreviations)

**Fig. 11:**
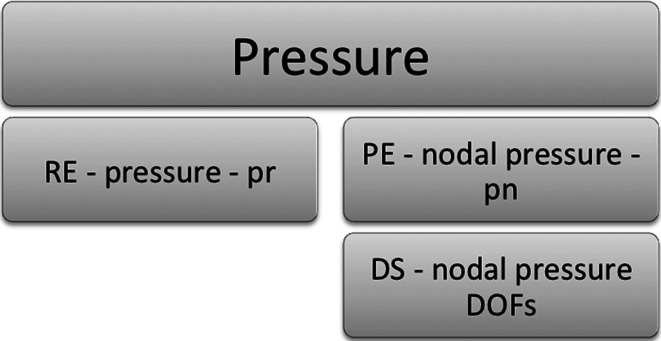
Detailed schematic for the pressure-specific part of the example model (see overview schematic for the explanation of abbreviations)

**Fig. 12:**
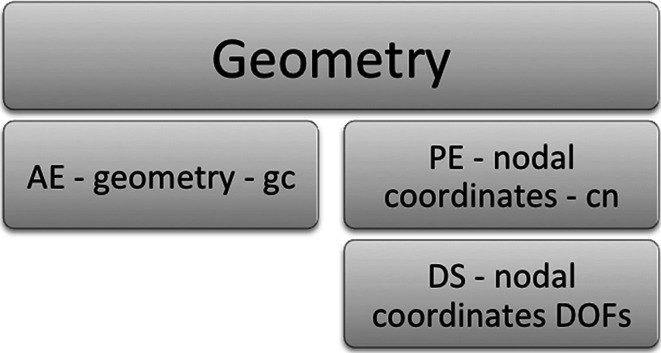
Detailed schematic for the geometry-specific part of the example model (see overview schematic for the explanation of abbreviations)

A schematic highlighting the main objects discussed is shown in Fig. 7, with details of the schematic shown larger in the figures that follow after it. The objects used in the FieldML model consist of those defined within the model file itself and those imported from the library. Here, only the objects from the library that are used by the model are of interest (Fig. 8). The objects defined in the model itself fall into three main categories: geometry, pressure and shared (Fig. 9). The objects that are shared relate to the aspects of the mesh that are shared by the geometry and pressure fields, such as the elements, their shapes and the local node to global node mapping that arises from the connectivity of the nodes (Fig. 10). The shared objects can be thought of as collectively defining a field template that will be used for the pressure and geometry fields. The pressure field is slightly simpler than the geometry field. It uses a reference evaluator to bind the pressure DOFs to the template (Fig. 11). The geometry field performs a similar binding, but needs to use an aggregate evaluator, since the geometry field is three dimensional, rather than just being scalar-valued (Fig. 12).

## Future work

FieldML 0.5 does not adequately represent topological structure or differential structure. Addressing this shortcoming is the first priority for the next version of FieldML. As mentioned earlier, FieldML 0.5 also cannot directly represent the arbitrary algebraic expressions needed to define interpolation methods, and it also lacks the ability to express PDE systems or, more generally, any implicit field descriptions. Design work to address these shortcomings is well advanced, and a prototype has already been created^27^ that can represent fields purely algebraically as well, supporting the FEM fields directly via an algebraic style. This prototype also allows for topological structure to be conveyed, but does not represent differential structure. However, this prototype relies on being embedded in the Haskell language. At the time of writing, progress has also been made on a second prototype that allows the testing of design ideas in a self-contained language.^28^ Both of these prototypes are based on designs that are still in the proposal stage, with prototyping being used to allow for more robust testing of the design ideas. A key issue to address is whether the algebraic approach to representing FieldML domains and fields can scale to meet the requirements of large high-performance computational simulation codes. Once the algebraic representation feature is available in FieldML, it will mark a significant capability that is not yet available in any comparable field representation data format.

An immediate goal for FieldML is to support a broader range of methods for describing FieldML domains, such as forming subsets of existing FieldML domains, which would be useful for metadata annotation of anatomical regions of interest in generic models, or regions of interest in individualised models such as regions of the myocardium with ischaemic damage. This is part of the top priority work being done for the next version of FieldML. A method for representing this is demonstrated in the aforementioned prototypes.

Nevertheless, a large number of FEM meshes and their associated fields can be effectively represented using FieldML version 0.5. Much work is needed to improve the adoption of FieldML, and this will largely depend on implementing FieldML I/O support in relevant software. An approach that has been tried on a small scale for the CellML format and API is to directly contribute code to open source third-party software projects that express an interest in supporting CellML. A similar approach may prove useful for FieldML too.

Although the number of models represented in the most up-to-date versions of the FieldML format is still relatively small, a large number of models exist in its predecessor formats, and these will be converted to FieldML and made available in the model repository in the future.

The FieldML API support for parallel I/O via HDF5 still needs to be validated, and it is expected that more comprehensive access to configuration options via the FieldML API will be needed so that fast I/O can be achieved for a wider variety of execution environments. Support for other underlying parallel I/O data format systems, such as NetCDF [35], is also likely in future. Work has already begun on implementing FieldML parallel I/O via parallel HDF5 in OpenCMISS, and using this for the I/O of distributed computations for FEM solution data will help guide this aspect of the FieldML design in the near future. Support for high-performance parallel I/O for large-scale problems is currently being treated as the second-highest-priority issue for the next version of FieldML.

Direct interchange of FieldML data between applications without having to first serialise this data is a feature of the API that is planned for the future. The ability to directly evaluate fields using the API is also likely to be incorporated into a future version of the API.

A current focus for CellML is the improvement of tools for annotation of CellML models [2], and this will also be an important feature of FieldML. For CellML, some progress towards this has been made by the development of the OpenCOR open source software.^29^

CellML has for a long time had comprehensive support for specifying the physical units used within a model, and this is a feature that will be needed in FieldML. CellML has always used algebraic expressions to describe the bulk of the model structure. Nevertheless, efficient computation is possible by means of code generation, which is the approach used by the CellML API. This approach may prove useful for processing FieldML, although the focus on supporting large models efficiently, especially on parallel computing architectures, will mean that fundamentally new approaches to code generation will be needed.

There is the potential for FieldML and CellML to be merged in the future, especially once FieldML has full support for representing both algebraic and differential equations, and once FieldML has support for physical units of measure on scalar-valued fields and scalar components of more complex fields. Indeed, as the development of standards such as CellML and FieldML progresses in future, we envisage that they will possibly eventually converge to a smaller number of more unified standards.

A key area of future work for PMR is integration with metadata standards and technologies. This will allow model authors to submit data to PMR that has been annotated and that the PMR system will then index. This will allow for more advanced methods of discovering models housed in PMR, and of presenting information about those models.

## Conclusion

FieldML is under active development; nevertheless, its design is based on well-established formats, and version 0.5 is already able to represent a wide range of models. Key innovations include the flexible composability of evaluators, the flexible referencing of intact external data sources and the facility to modularise and abstract field representations by means of external evaluators. The design of FieldML version 0.5, and perhaps more importantly the experience gained from creating this design during the euHeart project, also provides a strong foundation for the design of the next version, for which prototypes are already being developed.

The mathematical model representation challenge that CellML addresses is simpler than that for FieldML, and so it has been possible to bring the CellML standard and tools to a level of maturity, and we have attempted to apply some of the lessons learnt from developing CellML to the development of FieldML.

## Electronic supplementary material

Below is the link to the electronic supplementary material.

Supplementary material 1 (PDF 310 kb)

Supplementary material 2 (PDF 5792 kb)

Supplementary material 3 (PDF 3236 kb)

## Footnotes

1
www.euheart.eu.

2 See Sect. 5.1.

3
http://paraview.org/Wiki/ParaView:FAQ#What_file_formats_does_ParaView_support.3F.

4
https://wci.llnl.gov/codes/visit/FAQ.html#12.

5
http://www.generalmeshviewer.com.

6
http://www.hdfgroup.org/HDF5.

7
http://cgns.sourceforge.net/WhatIsCGNS.html.

8
http://cgns.sourceforge.net/hdf5.html.

9
http://www.vtk.org/VTK/img/file-formats.pdf.

10 In the context of FEM and FieldML, the term “element shape” refers to the intuitive geometric shape that one thinks of independent of the shape into which the element is deformed when it models part of the geometry of the overall FEM mesh. In other words, “element shape” simply means the geometric shape that a single finite element would have if the field describing its geometry was the identity field of its element coordinates.

11
http://www.grc.nasa.gov/WWW/cgns/CGNS_docs_current/sids/gridflow.html.

12
http://www.grc.nasa.gov/WWW/cgns/CGNS_docs_current/sids/dataname.html#dataname_grid.

13
http://models.cellml.org/exposure/a7179.

14 See Sects. 5.4 and 5.6.

15
https://amdb.isd.kcl.ac.uk:8443/AMDBWebInt/geometricalModelPage.do?gmId=153. Note that a username and a password are required to access this resource; this can be obtained from the website.

16 See Sect. 5.4.

17
http://www.openmath.org/standard/om20-2004-06-30/omstd20html-1.xml.

18
http://en.wikipedia.org/wiki/Serialization.

19
http://www.xmlsoft.org/.

20 See http://www.cmiss.org/cmgui. Cmgui is used within euHeart, mainly with the legacy format. The cmgui API is used by GIMIAS (http://www.gimias.net), another euHeart software project.

21 The term domain is used in two different ways in FieldML. Whenever the usage might be ambiguous, *FieldML domain* is used to refer to a set of points defined in FieldML, and *function domain* is used to refer to the set of possible inputs to a function (analogous to codomain being the set of possible outputs of a function).

22 See Sect. 5.7.

23 See the section “Future work”.

24 Composition here means mathematical function composition, as in $h = f \circ g$ means $h(x) = f(g(x)).$

25 Arbitrarily adding fields is not yet supported in FieldML version 0.5, although planned for future versions, so this is just illustrative.

26 Vector here is used without conveying that it is an element of a vector space with the accompanying algebra, but rather merely to indicate that it is an object that consists of collection of indexed scalar real values.

27
https://github.com/codecurve/FieldML-Haskell-01.

28
https://github.com/A1kmm/declarative-fieldml-prototype.

29
http://www.opencor.ws/.
