# Supplementary material for: FieldML, a proposed open standard for the Physiome project for mathematical model representation
Source: Med Biol Eng Comput. 2013 Jul 31;51(11):1191–207. doi: 10.1007/s11517-013-1097-7 (PMC3825639; doi:10.1007/s11517-013-1097-7)
Supplement: Supplementary file 1 — Supplementary material 1 (PDF 310 kb) [file 11517_2013_1097_MOESM1_ESM.pdf]

11 Appendix A – Technical detail of the FieldML 0.5 XML file format

This is one of the electronic supplementary material (ESM) items for the article "FieldML, a proposed open standard for the Physiome project for mathematical model representation".

Section 5 of the article described the FieldML 0.5 data model. This section describes the details of how XML is used to represent that data model. It is assumed that the reader is familiar with XML in general. A good introduction to XML is available from http://www.w3schools.com/xml/ and we do not reintroduce concepts and terminology here that are adequately covered in such texts. The general structure here is that for each tag, a table presents a summary of the XML structure of that tag, showing the possible attributes and child elements or other content, followed by a description, and an example if appropriate.

11.1 FieldML and Region elements

Tag:	`<Fieldml>`
Class:	None
Attributes:	`version, id`
Content:	`<Region>`

FieldML models are serialised in an XML format. The top-level element[1] has qualified name "FieldML", and has an attribute, version, which takes the value 0.5 to identify that the model is defined in FieldML 0.5.

The FieldML XML schema has been defined using XML Schema Definition; the defined XML schema is available at http://www.fieldml.org/resources/xml/0.5/FieldML_0.5.xsd.

FieldML elements always contain a single Region element.

Tag:	`<Region>`
Class:	None
Attributes:	`name, id`
Content:	`<EnsembleType>`<br>`<ContinuousType>`<br>`<MeshType>`<br>`<BooleanType>`<br>`<ArgumentEvaluator>`<br>`<PiecewiseEvaluator>`<br>`<ReferenceEvaluator>`<br>`<AggregateEvaluator>`<br>`<ExternalEvaluator>`

[1] As defined in the XML specification; XML elements should not be confused with finite elements.

	<ConstantEvaluator><br><ParameterEvaluator><br><Import><br><DataResource>

The Region element requires a name attribute, which may be any string. The region element does not serve a useful purpose in FieldML 0.5, but future versions of FieldML are likely to allow multiple Region elements in a FieldML element, at which point the region will act as a namespace, so that the same identifier may be defined differently in different parts of a file.

Many of the subsequently introduced XML elements require a name attribute. Name attribute values may be arbitrary strings of characters. However, by convention, they use a series of dot delimited parts to convey the hierarchy in which model components are organised. Within a given region element, FieldML has a global uniqueness rule: no two names may be the same. This rule applies even between completely different types of element (for example, it is not valid to define a data source and an argument evaluator with the same name). Similarly, many elements permit an "id" attribute, which can be any unique string within the model, and is intended as the target for RDF metadata annotations[2].

Example

The following example shows the utilisation of FieldML and Region:

```
<?xml version="1.0" encoding="UTF-8"?>
<Fieldml version="0.5"
      xmlns:xsi="http://www.w3.org/2001/XMLSchema-instance"
      xmlns:xlink="http://www.w3.org/1999/xlink"
      xsi:noNamespaceSchemaLocation=
            "http://www.fieldml.org/resources/xml/0.5/FieldML_0.5.xsd">
  <Region name="NAME">
        <!-- type, evaluator and other definitions here -->
  </Region>
</Fieldml>
```

11. 2 Defining ensemble types

Tag:	<EnsembleType>
Class:	Type
Attributes:	name, id
Content:	<Members>

Ensemble types may be defined by placing an EnsembleType element within the Region element. EnsembleType elements always have a name attribute, giving the name by which the ensemble type can be referred to.

[2] RDF is the Resource Description Framework, see http://www.w3.org/RDF/.

EnsembleType elements contain a single child element, Members[3].

Tag:	`<Members>`
Class:	None
Attributes:	
Content:	`<MemberRange>`<br>`<MemberListData>`<br>`<MemberRangeData>`<br>`<MemberStrideRangeData>`

The Members element in turn contains an element that defines the members of the ensemble. There are four such elements that may be included:

MemberRange, MemberListData, MemberRangeData, and MemberStrideRangeData.

Tag:	`<MemberRange>`
Class:	None
Attributes:	`max, min, stride`
Content:	None

The MemberRange element defines a sequence of ensemble members by a minimum label value (the min attribute), a maximum label value (the max attribute), and the difference between consecutive member labels (the stride attribute; the attribute defaults to 1 if omitted).

Tag:	`<MemberListData>`<br>`<MemberRangeData>`<br>`<MemberStrideRangeData>`
Class:	None
Attributes:	`count, data`
Content:	None

The remaining three ensemble membership definition elements (MemberListData, MemberRangeData, and MemberStrideRangeData) have two mandatory attributes: the count attribute, giving the number of members in the ensemble, and the data attribute, giving a reference to a data source. The interpretation of the data in the data source varies between the three elements.

The data source referenced from the MemberListData element contains a list of all valid member labels by enumeration.

---

[3] A future version of FieldML will likely remove the Members element, and its children will be direct children of the EnsembleType element.

The data source referenced from the MemberStrideRangeData element, on the other hand, arranges the data into a matrix with three columns (length 3). Each row of the data defines a minimum label, maximum label, and difference between consecutive members (stride). The ranges defined by each row must be non-overlapping. The data source referenced from the MemberRangeData element follows the same approach as for the MemberStrideRangeData, except that there are only two columns in the data source, and the stride is implicitly 1.

Example

The following example shows the utilisation of the EnsembleType element:

```
<!-- single range, optional stride defaults to 1 -->
<EnsembleType name="cube.nodes">
      <Members>
            <MemberRange min="1" max="8" stride="1" />
      </Members>
</EnsembleType>
```

The following more complex example (using data sources, which are introduced later in the manuscript) follows:

```
<!-- ranges with default stride of 1: 1 2 3 4 50 51 52 53 -->
<DataResource name="cube.nodes.range.resource">
      <DataResourceDescription>
            <DataResourceString>
                  1 4
                  50 53
            </DataResourceString>
      </DataResourceDescription>
      <ArrayDataSource name="cube.nodes.range.data" rank="2">
            <RawArraySize>2 2</RawArraySize>
      </ArrayDataSource>
</DataResource>

<EnsembleType name="cube.nodes">
      <Members>
            <MemberRangeData count="8" data="cube.nodes.range.data"/>
      </Members>
</EnsembleType>
```

11.3 Defining Boolean types

Tag:	`<BooleanType>`
Class:	Type
Attributes:	`name`

Content:	None

Boolean types may be defined by placing a BooleanType element within the Region element. The BooleanType element must have a name attribute, and does not have any other attributes or content.

Example

The standard library defines a Boolean type as follows:

```
<BooleanType name=”boolean”/>
```

11.4 Defining continuous types

Tag:	`<ContinuousType>`
Class:	Type
Attributes:	`name`
Content:	`<Components>`

Continuous types may be defined by placing a ContinuousType element within the Region element. The name attribute must be present. When defining a scalar type, no further elements or attributes are required.

When defining continuous vector types, a Components element must be added as a child of the ContinuousType element.

Tag:	`<Components>`
Class:	None
Attributes:	`name, count`
Content:	None

The Components element takes a name attribute, which is used to define an ensemble type (called the scalar index type) for referencing the dimensions of the continuous type. In addition, it takes a count attribute, which specifies the number of dimensions in the model.

Example

The following examples show the utilisation of the ContinuousType element:

```
<ContinuousType name="real.1d"/>
<ContinuousType name="chart.3d">
      <Components name="chart.3d.component" count="3"/>
</ContinuousType>
```

11.5 Defining mesh types

Tag:	`<MeshType>`

Class:	Type
Attributes:	`name`
Content:	`<Elements>`<br>`<Chart>`<br>`<Shapes>`

Mesh types may be defined by placing a MeshType element within the Region element. The MeshType element must have a name attribute, exactly one Elements element child, exactly one Chart element child, and exactly one Shapes element child.

The Elements element has the same requirements it would have if it was an EnsembleType element; it defines the ensemble part of the mesh exactly as if it had appeared as an EnsembleType child of the Region, except that the ensemble type name is formed from the name attribute on the MeshType element, followed by a period (.), followed by the name attribute on the Elements element.

The Chart element has the same requirements it would have if it was a ContinuousType element, and defines the chart part of the mesh exactly as if it had appeared as a ContinuousType child of the Region, except that the chart type name is formed from the name attribute on the MeshType element, followed by a period (.), followed by the name attribute on the Chart element.

The element and chart part of any argument having a mesh type can be accessed by suffixing the name of the argument with a period, followed by the name attribute on the Elements or Chart element, respectively.

Tag:	`<Shapes>`
Class:	None
Attributes:	`evaluator`
Content:	None

The Shapes element may take an attribute, evaluator, which must refer to an evaluator. This value gives the shape of all charts in the mesh. The evaluator attribute of the Shapes element must reference a Boolean-valued evaluator defined over the element chart and optionally also a mesh element argument. For constant shapes a standard shape evaluator from the FieldML library can be used. For varying shapes a piecewise evaluator can map different elements to different shape functions; for small meshes it may be practical to directly map the element index, but for large meshes it is more efficient to indirectly map the element index to a smaller shape ensemble and map these to shape functions.

Example

The following example shows the utilisation of the MeshType element:

```
<MeshType name="plate.mesh">
      <Elements name="elements">
```

```
            <Members>
                  <MemberRange min="1" max="4" />
            </Members>
      </Elements>
      <Chart name="chart">
            <Components name="plate.mesh.chart.component" count="2" />
      </Chart>
      <Shapes evaluator="heart.mesh.bounds" />
</MeshType>
```

11.6 Defining evaluators in general

Tag:	`<ArgumentEvaluator>`<br>`<ParameterEvaluator>`<br>`<PiecewiseEvaluator>`<br>`<AggregateEvaluator>`<br>`<ReferenceEvaluator>`<br>`<ExternalEvaluator>`
Class:	Evaluator
Attributes:	`name, valueType, id`
Content:	`<Arguments>`

Evaluators are defined by placing an element (the name of which will vary depending on the particular evaluator type) within the Region element. In addition to the attributes and child nodes defined on a per evaluator basis, every evaluator element must have a name attribute, giving a unique name to the evaluator, and a valueType attribute, naming the type to be produced by evaluation of the evaluator.

All evaluators except for ConstantEvaluators may optionally have a child element, Arguments, defining the inputs to the evaluator (note: while optional in general, this child attribute is mandatory for the ExternalEvaluator evaluator type).

Additionally, a Bindings child element is optional for three evaluator types: AggregateEvaluator, ReferenceEvaluator and PiecewiseEvaluator.

Tag:	`<Arguments>`
Class:	None
Attributes:	None
Content:	`<Argument>`

The Arguments element takes zero or more Argument element children.

Tag:	`<Argument>`

Class:	None
Attributes:	`name`
Content:	None

Each child Argument element must have a name attribute, naming an argument input to the evaluator.

Tag:	`<Bindings>`
Class:	None
Attributes:	None
Content:	`<Bind>`

Bindings elements have zero or more Bind element children.

Tag:	`<Bind>`
Class:	None
Attributes:	`argument, source`
Content:	None

Each Bind element must have an argument attribute, naming an ArgumentEvaluator, and representing the argument to bind to, and a source attribute, naming an evaluator, and representing the evaluator to be bound to the argument.

11.7 Defining argument evaluators

Tag:	`<ArgumentEvaluator>`
Class:	Evaluator
Attributes:	`name, valueType, id`
Content:	`<Arguments>`

Argument evaluators may be defined by placing an ArgumentEvaluator element within the Region element.

The only elements and attributes that can appear in an ArgumentEvaluator element are the standard ones applicable to all evaluator types.

Example

The following example shows how argument evaluators can be defined:

```
<ArgumentEvaluator name="cube.nodes.argument" valueType="cube.nodes"/>
```

In the following example, an argument evaluator defines its arguments explicitly to convey that it is a function over a given argument:

```
<ArgumentEvaluator name="cube.node.dofs.argument" valueType="real.type">
	<Arguments>
		<Argument name="cube.nodes.argument" />
	</Arguments>
</ArgumentEvaluator>
```

11.8 Defining parameter evaluators

Tag:	`<ParameterEvaluator>`
Class:	Evaluator
Attributes:	`name, valueType, id`
Content:	`<DenseArrayData>`<br>`<DOKArrayData>`<br>`<Arguments>`

Parameter evaluators may be defined by placing a ParameterEvaluator element within the Region element.

11.8.1 Dense parameters

In addition to the standard elements and attributes for all evaluator types, either a DenseArrayData or a DOKArrayData element must be present to define the data used to look up the value.

Tag:	`<DenseArrayData>`
Class:	None
Attributes:	`data`
Content:	`<DenseIndexes>`

A DenseArrayData element must have a data attribute, referring to a data source (discussed below), and a DenseIndexes element. The sequence of DenseIndexes elements defines the order in which the data should appear; the last DenseIndexes child element varies over a block of data, a series of these blocks are assembled by varying over the second to last index, and so on until a single block of data has been assembled.

Tag:	`<DenseIndexes>`
Class:	None
Attributes:	None
Content:	`<IndexEvaluator>`

The DenseIndexes element must contain a series of one or more IndexEvaluator elements

Tag:	<IndexEvaluator>
Class:	None
Attributes:	evaluator, order
Content:	None

IndexEvaluator must have an evaluator attribute, naming the ensemble-valued delegate evaluator that provides the value of the index. As a child of a DenseIndexes element, an IndexEvaluator element may optionally also have an order attribute, naming a data source that lists the ensemble elements in the order they are used to index the data.

11.8.2 Dense and sparse parameters

Tag:	<DOKArrayData>
Class:	None
Attributes:	keyData, valueData
Content:	<DenseIndexes><br><SparseIndexes>

The DOKArrayData element is used to define a data source using a sparse dictionary of keys formulation.

The DOKArrayData element may optionally have a DenseIndexes element (as described in section 0). The DOKArrayData element has two mandatory attributes, keyData and valueData, each of which must name a data source. The keyData data source contains a sequence of keys; each key contains one element for every sparse index, identifying the value of the sparse index to which the value applies. The keyData data source must be rank 2: first rank for each sparsely indexed value, second rank for the list of sparse index identifiers (even if there is only one sparse index). For each key, there is a value; a value consists of a block of data packed the same way as for DenseArrayData sources (or a single scalar if there is no DenseIndexes child on the DOKArrayData).

The DOKArrayData element must always have a SparseIndexes element child.

Tag:	<SparseIndexes>
Class:	None
Attributes:	None
Content:	<IndexEvaluator>

The SparseIndexes element must contain one or more IndexEvaluator elements.

Tag:	<IndexEvaluator>
Class:	None

Attributes:	`evaluator`
Content:	None

As a child of SparseIndexes, an IndexEvaluator element must have an evaluator attribute, naming the ensemble-valued evaluator that provides the value for the index.

<u>Example</u>

The following example shows how parameter evaluators can be defined, using an inline data resource (as defined later):

```
<DataResource name="grid.nodes.locations.resource">
      <DataResourceDescription>
            <DataResourceString>
                  <!-- X coords -->
                  0 0 0 0 0 0.25 0.25 0.25 0.25 0.25 0.5 0.5 0.5 0.5 0.5
0.75 0.75 0.75 0.75 0.75 1 1 1 1 1
                  <!-- Y coords -->
                  0 0.25 0.5 0.75 1 0 0.25 0.5 0.75 1 0 0.25 0.5 0.75 1 0
0.25 0.5 0.75 1 0 0.25 0.5 0.75 1
            </DataResourceString>
      </DataResourceDescription>
      <ArrayDataSource name="grid.nodes.locations.datasource" location="1"
rank="2">
            <RawArraySize>
                  2 25
            </RawArraySize>
      </ArrayDataSource>
</DataResource>
<ParameterEvaluator name="grid.nodes.nodalPositionByOrd"
valueType="real.1d">
      <DenseArrayData data="grid.nodes.locations.datasource">
            <DenseIndexes>
                  <IndexEvaluator
evaluator="coordinates.rc.2d.component.argument"/>
                  <IndexEvaluator evaluator="grid.nodes.argument"/>
            </DenseIndexes>
      </DenseArrayData>
</ParameterEvaluator>
```

11.9 Defining piecewise evaluators

Tag:	`<PiecewiseEvaluator>`
Class:	Evaluator
Attributes:	`name, valueType, id`
Content:	`<IndexEvaluators>`

	<EvaluatorMap><br><Arguments><br><Bindings>

Piecewise evaluators may be defined by placing a PiecewiseEvaluator element within the Region element. In addition to the standard elements and attributes for all evaluator types, an IndexEvaluators child element and an EvaluatorMap child element must be present.

Tag:	<IndexEvaluators>
Class:	None
Attributes:	None
Content:	<IndexEvaluator>

In FieldML 0.5, the IndexEvaluators element must always contain exactly one child element, IndexEvaluator.

Tag:	<IndexEvaluator>
Class:	None
Attributes:	evaluator, indexNumber
Content:	None

As a child of an IndexEvaluators element, an IndexEvaluator element must contain an evaluator attribute, referencing a delegate evaluator (the value type of which must be an ensemble type) and an indexNumber attribute, which must always have the value 1.

Tag:	<EvaluatorMap>
Class:	None
Attributes:	default
Content:	<EvaluatorMapEntry>

The EvaluatorMap element may have a "default" attribute whose value is the name of the delegate evaluator which defines the value of the piecewise at indices for which there is no matching EvaluatorMap. The delegate evaluator's value type must match the valueType of the piecewise evaluator. The EvaluatorMap element may also have N EvaluatorMapEntry child elements, where $0 \leq N \leq M$, and M is the cardinality of the ensemble referenced by the aforementioned IndexEvaluator.

Tag:	<EvaluatorMapEntry>

Class:	None
Attributes:	`value, evaluator`
Content:	None

Each EvaluatorMapEntry element must have both a value attribute (with a value equal to a member of the ensemble type of the index evaluator) and an evaluator argument (containing the name of the delegate evaluator which defines the value of the piecewise for the index value specified by the aforementioned “value” attribute). Again, the type of the delegate evaluator must match the valueType of the piecewise evaluator.

<u>Example</u>

The following example shows how piecewise evaluators can be defined:

```
<PiecewiseEvaluator name="plate.template.bilinear" valueType="real.type">
      <IndexEvaluators>
            <IndexEvaluator evaluator="plate.mesh.argument.elements"
indexNumber="1" />
      </IndexEvaluators>
      <ElementEvaluators>
            <ElementEvaluator indexValue="1"
evaluator="plate.bilinearLagrange"/>
            <ElementEvaluator indexValue="2"
evaluator="plate.bilinearSimplex"/>
            <ElementEvaluator indexValue="3"
evaluator="plate.bilinearSimplex"/>
            <ElementEvaluator indexValue="4"
evaluator="plate.bilinearLagrange"/>
      </ElementEvaluators>
</PiecewiseEvaluator>
```

This example also defines a piecewise evaluator, but makes use of a default evaluator:

```
<PiecewiseEvaluator name="cube.template.trilinear" valueType="real.type">
      <IndexEvaluators>
            <IndexEvaluator evaluator="cube.mesh.argument.elements"
indexNumber="1" />
      </IndexEvaluators>
      <ElementEvaluators default="cube.trilinear.interpolator" />
</PiecewiseEvaluator>
```

The following example combines the use of default with specified delegates, so that simplex interpolation is used for all elements other than elements 7 and 8, for which bilinear Lagrange interpolation is used.

```
<PiecewiseEvaluator name="plate.template.bilinear" valueType="real.type">
      <IndexEvaluators>
```

```
            <IndexEvaluator evaluator="plate.mesh.argument.elements"
indexNumber="1" />
      </IndexEvaluators>
      <ElementEvaluators default="plate.bilinearSimplex" >
            <ElementEvaluator indexValue="7"
evaluator="plate.bilinearLagrange"/>
            <ElementEvaluator indexValue="8"
evaluator="plate.bilinearLagrange"/>
      </ElementEvaluators>
</PiecewiseEvaluator>
```

11.10 Defining aggregate evaluators

Tag:	`<AggregateEvaluator>`
Class:	Evaluator
Attributes:	`name, valueType, id`
Content:	`<ComponentEvaluators>`<br>`<Arguments>`<br>`<Bindings>`

Aggregate evaluators may be defined by placing an AggregateEvaluator element within the Region element. In addition to the standard elements and attributes for all evaluator types, a ComponentEvaluators child element is required.

Tag:	`<ComponentEvaluators>`
Class:	None
Attributes:	`default`
Content:	`<ComponentEvaluator>`

The ComponentEvaluators element has a series of ComponentEvaluator child elements. There must either be a default evaluator provided via the “default” attribute, or there must be exactly one ComponentEvaluator element for each member of the index ensemble type referenced by the BindIndex argument evaluator value type.

Tag:	`<ComponentEvaluator>`
Class:	None
Attributes:	`component, evaluator`
Content:	None

The member of the index ensemble to which the component evaluator relates is specified by the mandatory component attribute. In addition, the mandatory evaluator attribute names the delegate argument evaluator to be used to evaluate the component of the vector.

Tag:	`<BindIndex>`
Class:	None
Attributes:	`indexNumber, argument`
Content:	None

If the optional Bindings element is present as a child of the AggregateEvaluator element, then it must contain a BindIndex child element. The BindIndex child element has a mandatory argument attribute, which names the delegate argument evaluator over which aggregation is to take place (that is, the index of the vector produced by aggregation). The value type of the argument evaluator must be an ensemble type.

The BindIndex element is required to have an indexNumber element, which in FieldML 0.5 always has the value 1.

Example

This example defines an aggregate evaluator over each component of a three dimensional reference co-ordinate system:

```
<AggregateEvaluator name="heart.coordinates" valueType="coordinates.rc.3d">
      <Bindings>
            <BindIndex argument="coordinates.rc.3d.component"
indexNumber="1"/>
            <Bind argument="heart.nodal_dofs"
source="heart.node.coordinates"/>
      </Bindings>
      <ComponentEvaluators>
            <ComponentEvaluator component="1"
evaluator="heart.template.triquadratic" />
            <ComponentEvaluator component="2"
evaluator="heart.template.triquadratic" />
            <ComponentEvaluator component="3"
evaluator="heart.template.biquadratic_linear" />
      </ComponentEvaluators>
</AggregateEvaluator>
```

11.11 Defining reference evaluators

Tag:	`<ReferenceEvaluator>`
Class:	Evaluator
Attributes:	`name, valueType, id`
Content:	`<Arguments>`<br>`<Bindings>`

A reference evaluator may be defined by placing a ReferenceEvaluator element within the Region element. In addition to the standard attributes for evaluators, the ReferenceEvaluator element must have an evaluator argument, which must name an ExternalEvaluator.

11.12 Defining external evaluators

Tag:	`<ExternalEvaluator>`
Class:	Evaluator
Attributes:	`name, valueType, id`
Content:	`<Arguments>`

External evaluators may be defined by placing an ExternalEvaluator element in the Region element. Other than the standard attributes and elements for evaluators, no additional attributes or child elements are required, but the standard Arguments child element is required to be present, to specify the parameters taken by the external evaluator.

External evaluators cannot be directly referenced as delegate evaluators, and so ReferenceEvaluators are used, referencing the external evaluator.

Example

The following example defines an external evaluator, and a reference evaluator that refers to it (note: normal practice is to define external evaluators in a library and import the name using the mechanism discussed below):

```
<ExternalEvaluator name="interpolator.3d.unit.trilinearLagrange"
valueType="real.1d">
      <Arguments>
            <Argument name="chart.3d.argument"/>
            <Argument
name="parameters.3d.unit.trilinearLagrange.argument"/>
      </Arguments>
</ExternalEvaluator>
<ReferenceEvaluator name="cube.trilinear.interpolator"
evaluator="interpolator.3d.unit.trilinearLagrange" valueType="real.1d">
      <Bindings>
            <Bind argument="chart.3d.argument"
source="cube.mesh.argument.chart" />
            <Bind argument="trilinearLagrange.parameters.argument"
source="cube.trilinearLagrange.parameters" />
      </Bindings>
</ReferenceEvaluator>
```

11.13 Defining constant evaluators

Tag:	`<ConstantEvaluator>`
Class:	Evaluator

Attributes:	`name, valueType, value, id`
Content:	None

The constant evaluator value tag simply contains a string representation of the value of that evaluator, as shown in the following example.

Example

```
<ConstantEvaluator name="name1" value="55" valueType="Ensemble1" />
```

11.14 Defining data resources and data sources

A data resource is a reference to some block of data. This block of data may be embedded in the FieldML XML file itself, or may occur in an external file referenced by the FieldML.

Tag:	`<DataResource>`
Class:	None
Attributes:	`name`
Content:	`<DataResourceDescription>`<br>`<ArrayDataSource>`

A data resource is declared by placing a DataResource element inside a Region element. Every DataResource element must have a name attribute; this name is not currently used for anything within FieldML, but may be used to refer to the DataResource from external applications.

The DataResource element must have at least one child element, which is either a DataResourceDescription element or an ArrayDataSource element.

Each DataResource element may define one or more data sources (which are referenced by ParameterEvaluators, as defined in section 0), by including an ArrayDataSource child element.

Tag:	`<DataResourceDescription>`
Class:	None
Attributes:	`name`
Content:	`<DataResourceHref>`<br>`<DataResourceString>`

The DataResourceDescription element contains another element, describing how to obtain the contents of the data resource. This may be either DataResourceHref or DataResourceString.

Tag:	`<DataResourceHref>`
Class:	None

Attributes:	xlink:href, format
Content:	None

DataResourceHref describes a reference to an external file. It has an xlink:href attribute (that is, an href attribute in the namespace http://www.w3.org/1999/xlink), using XLink. It additionally has a format attribute; supported values for the attribute are HDF5 and PLAIN_TEXT, indicating the data resource is represented in HDF5 or plain text, respectively.

Tag:	<DataResourceString>
Class:	None
Attributes:	xlink:href, format
Content:	Text representation of numerical data

The DataResourceString element allows the data to be contained directly within the XML file itself. The DataResourceString simply contains the text of the data resource.

Tag:	<ArrayDataSource>
Class:	None
Attributes:	location, name, rank
Content:	<RawArraySize><br><ArrayDataSize><br><ArrayDataOffset>

An ArrayDataSource element must have a name attribute, giving the data source a name it can be referred to by, a location attribute, giving an offset into a string or plain text href data source, or the path in an HDF5 data source, and a rank attribute, giving the dimensionality of the data source (i.e. the number of indices a parameter evaluator referring to the data source should have). The ArrayDataSource element may have a RawArraySize child element, and may also have an ArrayDataOffset element and an ArrayDataSize element.

Tag:	<RawArraySize><br><ArrayDataSize><br><ArrayDataOffset>
Class:	None
Attributes:	None
Content:	Text representation of numerical data

Each of these three elements must, if present, contain a series of integers, one for each dimension, with the number of integers matching the rank attribute. The integers in the RawArraySize element specify the number of entries in the data for each dimension. The entries in the ArrayDataOffset and ArrayDataSize specify the range within those elements that are actually used (as an offset and a size) within this particular data source.

11.15 Defining imports

Tag:	`<Import>`
Class:	None
Attributes:	`xlink:href, region`
Content:	`<ImportEvaluator>` `<ImportType>`

An import may be defined by placing an Import child element in the Region element. Every Import element must have a href attribute in the namespace http://www.w3.org/1999/xlink, giving the name of the FieldML file from which to import. In addition, the import element must have a region attribute, which must match the name of the Region element in the imported model.

The Import element may have ImportType and ImportEvaluator children, defining an import of a type or an evaluator, respectively.

Tag:	`<ImportEvaluator>` `<ImportType>`
Class:	None
Attributes:	`localName, remoteName`
Content:	None

ImportType and ImportEvaluator elements must have two attributes, localName, giving the name to make the imported type or evaluator available as in the importing model, and remoteName, giving the name of the type or evaluator in the imported model.

The localNames will be available to use as a type or evaluator name exactly as if they were directly defined as a type or evaluator in the importing model.

Importing a name does not make a copy or instance of the underlying object; instead, each import refers to the same underlying object. For example, if two models import the same type from another model, each define an evaluator of that type, and then another model in turn imports the two evaluators from the two models, the value types of the two evaluators will be identical, even if the same type was imported under different names in the different models.

Example

The following example shows how an import can be defined:

```
<Import
xlink:href="http://www.fieldml.org/resources/xml/0.5/FieldML_Library_0.5.xm
l"
region="library">
	<ImportType localName="real.type" remoteName="real.1d" />
	<ImportType localName="triquadraticSimplex.parameters"
remoteName="parameters.3d.unit.triquadraticSimplex" />
	<ImportEvaluator localName="chart.3d.argument"
```

```
remoteName="chart.3d.argument" />
      <ImportEvaluator localName="triquadraticSimplex.parameters.argument"
remoteName="parameters.3d.unit.triquadraticSimplex.argument" />
      <ImportEvaluator localName="triquadraticSimplex.interpolator"
remoteName="interpolator.3d.unit.triquadraticSimplex" />
</Import>
```

11.16 The FieldML standard Library details

The FieldML 0.5 standard library was introduced in section 5.7 of the article. This section describes the contents of the library, and the meaning of the external evaluators used in the FieldML 0.5 standard library.

The types real.1d, real.2d, and real.3d define one, two, and three-dimensional real types, and are used extensively in the FieldML standard library. The two and three-dimensional charts have a component type defined under the names real.2d.component and real.3d.component, respectively.

In exactly the same way, a corresponding set of types name chart.1d, chart.2d, and so on are defined to refer to chart co-ordinates, and coordinates.rc.1d, coordinates.rc.2d, and so on, to refer to reference co-ordinates.

A series of shapes are defined as evaluators that map from a co-ordinate space to a Boolean value are defined. These can be used with the Shapes element to define shapes in element meshes. The following table shows the shapes defined in the standard library.

Table 1 Shapes in the FieldML 0.5 standard library.

Shape name	Description	Definition
`shape.unit.line`	1-D unit line	$x \in [0,1]$
`shape.unit.square`	2-D unit square	$x_1 \in [0,1] \wedge x_2 \in [0,1]$
`shape.unit.triangle`	2-D triangle	$x_1 \geq 0 \wedge x_2 \geq 0 \wedge x_1 + x_2 \leq 1$
`shape.unit.cube`	3-D unit cube	$x_1 \in [0,1] \wedge x_2 \in [0,1] \wedge x_3 \in [0,1]$
`shape.unit.tetrahedron`	3-D tetrahedron	$x_1 \geq 0 \wedge x_2 \geq 0 \wedge x_3 \geq 0 \wedge x_1 + x_2 + x_3 \leq 1$
`shape.unit.wedge12`	Wedge	$x_1 \geq 0 \wedge x_2 \geq 0 \wedge x_1 + x_2 \leq 1 \wedge x_3 \in [0,1]$
`shape.unit.wedge23`	Wedge	$x_3 \geq 0 \wedge x_2 \geq 0 \wedge x_3 + x_2 \leq 1 \wedge x_1 \in [0,1]$
`shape.unit.wedge13`	Wedge	$x_1 \geq 0 \wedge x_3 \geq 0 \wedge x_1 + x_3 \leq 1 \wedge x_2 \in [0,1]$

Next, a series of external evaluators, for interpolators, and types, for the parameters to those interpolators, are defined. The interpolators are defined in the form interpolator.nd.unit.interpolatorName, where n in “nd” is replaced with a dimension (either 1, 2, or 3 in the standard library), and interpolatorName is the name of the interpolators.

The following table shows the available interpolators; for example, taking parts from the different columns, the table shows that there is an interpolator called interpolator.3d.unit.tricubicHermite. This interpolator is a standard unit tri-cubic Hermite. For each such external evaluator, a parameter type with the same name is defined, but with interpolator substituted for parameters (for example, parameters.3d.unit.tricubicHermite).

Table 2 Interpolators in the standard library

Dimension / Type	Lagrange	Hermite	Hermite - scaled	Simplex
1d	Linear			
	Quadratic			
	Cubic	Cubic	Cubic	
2d	Bilinear			Bilinear
	Biquadratic			Biquadratic
	Bicubic	Bicubic	Bicubic	
3d	Trilinear			Trilinear
	Triquadratic			Triquadratic
	Tricubic	Tricubic	Tricubic	

However, for the HermiteScaled evaluators, the same parameters type is used as for the corresponding unscaled Hermite evaluator, but with an additional argument describing the scaling; a type is defined for these arguments, in a form like parameters.3d.unit.tricubicHermiteScaling. Note that in addition to those defined in the table, interpolator.3d.unit.triquadraticSimplex.zienkiewicz is available for Zienkiewicz triquadratic simplex interpolation [42].

In addition, ensemble types are defined to identify each local nodal point used by each interpolator as follows: For Lagrange and Hermite interpolators, localNodes.1d.line*n* is defined, where *n* is 2 for the linear case, 3 for the quadratic case, and 4 for the cubic case; localNodes.2d.square*nxn* is defined at n in {2; 3; 4} for the bilinear, biquadratic, and bicubic cases, and localNodes.3d.cube*nxnxn* is defined at n in {2; 3; 4} for the trilinear, triquadratic, and tricubic cases. For the simplex method, localNodes.2d.triangle3, localNodes.2d.triangle6, localNodes.3d.tetrahedron4, and localNodes.3d.tetrahedron10 are defined for the bilinear simplex, biquadratic simplex, trilinear simplex and triquadratic simplex cases, respectively.

For each type in the library, and argument evaluator is defined with the name of the type followed by .argument; these argument evaluators are used as the inputs to the external evaluators.
