# Supplementary material for: FieldML, a proposed open standard for the Physiome project for mathematical model representation
Source: Med Biol Eng Comput. 2013 Jul 31;51(11):1191–207. doi: 10.1007/s11517-013-1097-7 (PMC3825639; doi:10.1007/s11517-013-1097-7)
Supplement: Supplementary file 2 — Supplementary material 2 (PDF 5792 kb) [file 11517_2013_1097_MOESM2_ESM.pdf]

12 Supplementary material - A simple example with a line-by-line description

12.1 XML listing of FieldML example

Listing 1 The FieldML version 0.5 XML listing for the example from section 6, for a simple mesh of 4 elements. The FieldML file is available online at http://models.physiomeproject.org/w/randall/FieldML-example-simple-mesh. This is based on a single element example available at http://cmiss.bioeng.auckland.ac.nz/development/examples/a/a1/cmiss_input/cube.xml.

```xml
<?xml version="1.0" encoding="UTF-8"?>
<Fieldml version="0.5"
  xmlns:xsi="http://www.w3.org/2001/XMLSchema-instance"
  xmlns:xlink="http://www.w3.org/1999/xlink"
  xsi:noNamespaceSchemaLocation="http://www.fieldml.org/resources/xml/0.5/FieldML_0.5.xsd">
<Region name="mesh1">

<!-- make types and evaluators from the library visible in this region
  under local names -->
<Import xlink:href="http://www.fieldml.org/resources/xml/0.5/FieldML_Library_0.5.xml" region="library">
  <ImportType localName="real.type" remoteName="real.1d" />
  <ImportType localName="trilinearLagrange.parameters" remoteName="parameters.3d.unit.trilinearLagrange" />
  <ImportType localName="trilinearLagrange.points" remoteName="parameters.3d.unit.trilinearLagrange.component" />
  <ImportType localName="coordinates.rc.3d.component" remoteName="coordinates.rc.3d.component" />
  <ImportType localName="coordinates.rc.3d" remoteName="coordinates.rc.3d" />
  <ImportEvaluator localName="trilinearLagrange.parameters.argument" remoteName="parameters.3d.unit.trilinearLagrange.argument" />
  <ImportEvaluator localName="trilinearLagrange.points.argument" remoteName="parameters.3d.unit.trilinearLagrange.component.argument" />
  <ImportEvaluator localName="trilinearLagrange.interpolator" remoteName="interpolator.3d.unit.trilinearLagrange" />
  <ImportEvaluator localName="chart.3d.argument" remoteName="chart.3d.argument" />
  <ImportEvaluator localName="coordinates.rc.3d.component.argument" remoteName="coordinates.rc.3d.component.argument" />
  <ImportEvaluator localName="shape.unit.cube" remoteName="shape.unit.cube"/>
</Import>

<!-- define 20-member ensemble to represent nodes 1..20 -->
<EnsembleType name="mesh1.nodes">
  <Members>
    <MemberRange min="1" max="20" />
  </Members>
</EnsembleType>


<!-- declare an argument of nodes type; this represents a value source
  that other evaluators can map their values to -->
<ArgumentEvaluator name="mesh1.nodes.argument" valueType="mesh1.nodes" />

<!-- declare an argument representing the node-indexed DOFs for a field
```

```
  template, a real value expected to be a function of "mesh1.nodes.argument" -->
<ArgumentEvaluator name="mesh1.node.dofs.argument" valueType="real.type">
  <Arguments>
    <Argument name="mesh1.nodes.argument" />
  </Arguments>
</ArgumentEvaluator>

<!-- define a 3-D mesh type with 4 elements, each of unit cube shape -->
<MeshType name="mesh1.mesh.type">
  <Elements name="elements">
    <Members>
      <MemberRange min="1" max="4" />
    </Members>
  </Elements>
  <Chart name="chart">
    <Components name="mesh1.mesh.type.chart.component" count="3" />
  </Chart>
  <Shapes evaluator="shape.unit.cube"/>
</MeshType>

<!-- declare an argument of the mesh type. Evaluators varying only with this
  argument are interpreted as fields over the mesh -->
<ArgumentEvaluator name="mesh1.mesh.argument" valueType="mesh1.mesh.type" />

<!-- An inline data resource listing 8 corner nodes per element. Resources
  are just raw data; a ParameterEvaluator is needed to add semantic meaning -->
<DataResource name="mesh1.nodes.connectivity.resource">
  <DataResourceDescription>
    <DataResourceString>
        1 2 3 4 5 6 7 8
        5 6 7 8 9 10  11  12
        9 10  11  12  13  14  15  16
        13  14  15  16  17  18  19  20
    </DataResourceString>
  </DataResourceDescription>
  <ArrayDataSource name="mesh1.nodes.connectivity.data" location="1" rank="2">
    <RawArraySize>
      4 8
    </RawArraySize>
  </ArrayDataSource>
</DataResource>

<!-- define mapping from element × localnode to global index from ensemble
  "mesh1.nodes.argument". "trilinearLagrange.points" are documented
  as being the 8 corner points of a unit cube at chart locations:
  (0,0,0), (1,0,0), (0,1,0), (1,1,0), (0,0,1), (1,0,1), (0,1,1), (1,1,1) -->
<ParameterEvaluator name="mesh1.trilinearLagrange.connectivity" valueType="mesh1.nodes">
  <DenseArrayData data="mesh1.nodes.connectivity.data">
```

```
    <DenseIndexes>
      <IndexEvaluator evaluator="mesh1.mesh.argument.elements" /><!-- Needs improvement: mesh1.mesh.arguments is an alias to mesh1.mesh.type which has an elements factor so this is a way of referring to that factor, using contents of the string. -->
      <IndexEvaluator evaluator="trilinearLagrange.points.argument" />
    </DenseIndexes>
  </DenseArrayData>
</ParameterEvaluator>

<!-- construct a vector of node parameters to pass on to
  "mesh1.trilinear.interpolator" -->
<AggregateEvaluator name="mesh1.trilinearLagrange.parameters"
    valueType="trilinearLagrange.parameters">
  <Bindings>
    <BindIndex argument="trilinearLagrange.points.argument" indexNumber="1" />
    <Bind argument="mesh1.nodes.argument" source="mesh1.trilinearLagrange.connectivity" />
  </Bindings>
  <ComponentEvaluators default="mesh1.node.dofs.argument" />
</AggregateEvaluator>

<!-- define evaluator returning value of library trilinear Lagrange interpolator
  at the element chart location of mesh type "mesh1.mesh" and using parameters
  from evaluator "mesh1.trilinearLagrange.parameters". -->
<ReferenceEvaluator name="mesh1.trilinear.interpolator"
    evaluator="trilinearLagrange.interpolator" valueType="real.type">
  <Bindings>
    <Bind argument="chart.3d.argument" source="mesh1.mesh.argument.chart" />
    <Bind argument="trilinearLagrange.parameters.argument" source="mesh1.trilinearLagrange.parameters" />
  </Bindings>
</ReferenceEvaluator>

<!-- define a piecewise template delegating which evaluator gives the template
  its values in each element, which is trivial for this one element mesh.
  It is a template for a field defined over the mesh represented by
  "mesh1.mesh.argument", with the unbound parameter source
  "mesh1.node.dofs.argument" inherited from delegate evaluator
  "cube.trilinear.interpolator" -->
<PiecewiseEvaluator name="mesh1.template.trilinear" valueType="real.type">
  <IndexEvaluators>
    <IndexEvaluator evaluator="mesh1.mesh.argument.elements" indexNumber="1" />
  </IndexEvaluators>
  <EvaluatorMap default="mesh1.trilinear.interpolator" />
</PiecewiseEvaluator>

<!-- inline data resource listing raw values for the 8 nodes * 3 components
  of the 'coordinates' field. ParameterEvaluator "mesh1.node.coordinates"
  gives the data semantic meaning. -->
<DataResource name="mesh1.coordinates.resource">
```

```
  <DataResourceDescription>
    <DataResourceString>
      0.0 0.0 0.0
      1.0 0.0 0.0
      0.0 1.0 0.0
      1.0 1.0 0.0
      0.0 0.0 1.0
      1.0 0.0 1.0
      0.0 1.0 1.0
      1.0 1.0 1.0
      0.0 0.0 2.0
      1.0 0.0 2.0
      0.0 1.0 2.0
      1.0 1.0 2.0
      0.0 0.0 3.0
      1.0 0.0 3.0
      0.0 1.0 3.0
      1.0 1.0 3.0
      0.0 0.0 4.0
      1.0 0.0 4.0
      0.0 1.0 4.0
      1.0 1.0 4.0
    </DataResourceString>
  </DataResourceDescription>
  <ArrayDataSource name="mesh1.coordinates.data" location="1" rank="2">
    <RawArraySize>
      20 3
    </RawArraySize>
  </ArrayDataSource>
</DataResource>

<!-- parameters for the coordinate field, listing a scalar real parameter
  for all permutations of library 3-component ensemble
  "coordinates.rc.3d.component" and 8-member ensemble "mesh1.nodes.argument" -->
<ParameterEvaluator name="mesh1.node.coordinates" valueType="real.type">
  <DenseArrayData data="mesh1.coordinates.data">
    <DenseIndexes>
      <IndexEvaluator evaluator="mesh1.nodes.argument" />
      <IndexEvaluator evaluator="coordinates.rc.3d.component.argument" />
    </DenseIndexes>
  </DenseArrayData>
</ParameterEvaluator>

<!-- define the final vector coordinates field by aggregating evaluators for
  each component of the vector valueType. Although each component uses the
  same evaluator in this example, they produce different values because the
  parameters on which they depend vary with the same component ensemble
  argument ("coordinates.rc.3d.component.argument"). -->
```

```
<AggregateEvaluator name="coordinates" valueType="coordinates.rc.3d">
  <Bindings>
    <BindIndex argument="coordinates.rc.3d.component.argument" indexNumber="1" />
    <Bind argument="mesh1.node.dofs.argument" source="mesh1.node.coordinates" />
  </Bindings>
  <ComponentEvaluators default="mesh1.template.trilinear" />
</AggregateEvaluator>

<!-- inline data resource listing pressure values for the 20 nodes. -->
<DataResource name="mesh1.pressure.resource">
  <DataResourceDescription>
    <DataResourceString>
       80000.0
      100000.0
      100000.0
       80000.0
      100000.0
       80000.0
       80000.0
      100000.0
       70000.0
      110000.0
      100000.0
       70000.0
      120000.0
       80000.0
       80000.0
       90000.0
       90000.0
      100000.0
      100000.0
       90000.0
    </DataResourceString>
  </DataResourceDescription>
  <ArrayDataSource name="mesh1.pressure.data" location="1" rank="1">
    <RawArraySize>
      20
    </RawArraySize>
  </ArrayDataSource>
</DataResource>

<!-- parameters for the pressure field, listing a scalar real parameter
  for each member of "mesh1.nodes.argument" -->
<ParameterEvaluator name="mesh1.node.pressure" valueType="real.type">
  <DenseArrayData data="mesh1.pressure.data">
    <DenseIndexes>
      <IndexEvaluator evaluator="mesh1.nodes.argument" />
    </DenseIndexes>
```

```
227     </DenseArrayData>
228   </ParameterEvaluator>
229
230   <!-- define a scalar 'pressure' field using the same template as
231     components of coordinates field -->
232   <ReferenceEvaluator name="pressure" evaluator="mesh1.template.trilinear" valueType="real.type">
233     <Bindings>
234       <Bind argument="mesh1.node.dofs.argument" source="mesh1.node.pressure" />
235     </Bindings>
236   </ReferenceEvaluator>
237
238   </Region>
239   </Fieldml>
```

12.2 Line-by-line description of XML example

The following describes in detail the meaning of each section of the XML example given in section 12.1. Throughout this section, shorter mathematical variable names will be used to allow brief mathematical equations that describe the FieldML to be written, rather than the identifiers used for the XML elements.

Line 1 is a standard XML preamble. Lines 2-5 make up the parent FieldML tag, the version attribute specifies the FieldML format version. Line 3 declares the XML “xsi” namespace for the attribute on line 5, essentially meaning that XML tags in this file that are not prefixed by a namespace are in the FieldML 0.5 schema defined by the FieldML 0.5 XML schema document at the given URL. Line 4 declares the “xlink” namespace so that XLink XML tags and attributes can be used. On line 9, XLink is used for the import, specifying the FieldML 0.5 standard library URL. Line 6 declares the “mesh1” region.

Line 11 imports the library “real.1d”, which in the library is declared as

```
<ContinuousType name="real.1d"/>
```

This is just $\mathbb{R}$, the standard mathematical set of real numbers.

Line 12 imports the type that represents a vector of length 8 that make up the DOF values used for tri-linear Lagrange FEM interpolation. This is declared in the library as

```
<ContinuousType name="parameters.3d.unit.trilinearLagrange">
    <Components name="parameters.3d.unit.trilinearLagrange.component"
count="8"/>
  </ContinuousType>
```

Line 13 imports the type that represents the index into the above vector, below, the symbol $LL3c$. Thus we have

$$LL3c = \{1,2,\ldots,8\}$$

These are essentially just the 8 local nodes of each FEM element, due to the nature of tri-linear Lagrange interpolation, so the local name chosen is “trilinearLagrange.points”. This illustrates how imports allow an arbitrary local name to be chosen. The XML for the target entity in the library is given in the above XML snippet, since the Components XML element names the index as part of the declaration of a multidimensional ContinuousType element.

It should also be pointed out that there are two notations for representing a vector of 8 real numbers in this situation. Firstly, as the set, $\mathbb{R}^8$, which is more common mathematical notation. Secondly as a function whose domain is $LL3c$, and whose codomain is $\mathbb{R}$

$$v2: LL3c \to \mathbb{R}$$

Here, $v2$ is an arbitrary variable representing the required vector like object. Using the first option, we might write $v1 \in \mathbb{R}^8$. Both $v1$ and $v2$ essentially represent the same information. The second notation includes additional information about how the members of the vector are indexed, whereas the first notation is more compact. We will refer to this set as $LL3p$.

$$LL3p = \mathbb{R}^8 = (LL3c \to \mathbb{R})$$

Skipping ahead, the imports on lines 16 and 17 can be thought of as taking variable declarations in the library and aliasing those locally. These variables are of the types from the imports on lines 12 and 13.

The XML for these in the library is

```
<ArgumentEvaluator name="parameters.3d.unit.trilinearLagrange.argument"
valueType="parameters.3d.unit.trilinearLagrange"/>
  <ArgumentEvaluator
name="parameters.3d.unit.trilinearLagrange.component.argument"
valueType="parameters.3d.unit.trilinearLagrange.component"/>
```

Line 18 imports the basis interpolator function that is an external evaluator in the library, and will be referred to in this description as $LL3$.

```
<ExternalEvaluator name="interpolator.3d.unit.trilinearLagrange"
valueType="real.1d">
    <Arguments>
      <Argument name="chart.3d.argument"/>
      <Argument name="parameters.3d.unit.trilinearLagrange.argument"/>
    </Arguments>
  </ExternalEvaluator>
```

In the above, “chart.3d.argument” refers to an argument evaluator declared in the library as

```
<ContinuousType name="chart.3d">
    <Components name="chart.3d.component" count="3"/>
  </ContinuousType>
  <ArgumentEvaluator name="chart.3d.component.argument"
valueType="chart.3d.component"/>
```

The corresponding argument evaluator of this type is also imported on line 19, the library declaration is

```
<ArgumentEvaluator name="chart.3d.argument" valueType="chart.3d"/>
```

This argument will be referred to here as ξ. Thus,

$$\xi \in \mathbb{R}^3$$

Thus the function domain of $LL3$ is the Cartesian product $\mathbb{R}^3 \times LL3p$. The codomain of $LL3$ is $\mathbb{R}$.

Hence we have:

$$LL3: \mathbb{R}^3 \times LL3p \rightarrow \mathbb{R}$$

Coming back to line 14, it imports the type used to index the components of a vector in 3D Cartesian space, $\mathbb{R}^3$, and $\mathbb{R}^3$ is itself imported on Line 15. The XML both of these in the library is

```
<ContinuousType name="coordinates.rc.3d">
    <Components name="coordinates.rc.3d.component" count="3"/>
  </ContinuousType>
```

And line 20 imports an argument evaluator of the type of the index into an $\mathbb{R}^3$ vector.

Line 21 imports a Boolean valued external evaluator, which will be used as the predicate define $U3$, which will be referred to here as $pU3$. The XML in the library for it is

```
<ExternalEvaluator name="shape.unit.cube" valueType="boolean">
    <Arguments>
      <Argument name="chart.3d.argument"/>
    </Arguments>
  </ExternalEvaluator>
```

Using mathematical notation to define the domain and codomain of $pU3$ is done as follows:

$$pU3: \mathbb{R}^3 \rightarrow \mathbb{B}$$

Here $\mathbb{B}$ represents the set corresponding to the Boolean type.

Lines 25 to 29 declare an ensemble, for mesh1's global nodes, i.e. a discrete set

$$m1n = \{1,2, \ldots ,20\}$$

Line 34 can be thought of as declaring a variable $m1na$ representing a value from the set $m1n$.

Using standard mathematical set theory notation, this is written as

$$m1na \in m1n$$

Line 38 to 42 declares

$$m1dof: m1n \rightarrow \mathbb{R}$$

This function is used to obtain a scalar nodal value at each global node of the mesh.

Lines 45 to 55 declare a FieldML mesh domain. The discrete set of FEM element identifiers is declared within the mesh declaration, lines 47-49, and it is just the set

$$m1e = \{1, 2, \ldots, 4\}$$

This mesh's structure is relatively simple since all elements are the same shape, so we can describe it using a simple Cartesian product, but this is not possible for meshes in general. Thus we have

$$m1 = m1e \times U3$$

On line 54, we have the unit cube defined as the subset using the predicate $pU3$:

$$U3 = [0,1] \times [0,1] \times [0,1]$$

Lines 63-77 provide the local to global node mapping. To make the file easier for humans to read, the data source string is laid out as one line per FEM element, providing global node numbers for each of the element local nodes in sequence. Nevertheless, FieldML processing software ignores this layout. It is on line 72 that the rank is specified to be 2, meaning that the data is indexed by two discrete indexes. And, the dimensions provided on line 74 convey the range of each index. Thus this is the same as a 4×8 matrix with integer entries, but it is easy to see how multidimensional arrays of dimension higher than 2 can be described.

A ParameterEvaluator declaration like that on Lines 83-90 can be thought of a wrapping multidimensional array whose indexes are just integer values into a more formal structure, where the indexes are ensembles, which are just discrete sets. The DOF mapping is informed by the intended connectivity. Although this evaluator's name is suffixed "connectivity", FieldML 0.5 does not formally have a way of representing topological connectivity. Thus connectivity is incidental to the FEM interpolation of the geometric coordinate field. Letting $m1c$ be the mathematical representation of this parameter evaluator is, we have

$$m1c: m1e \times LL3c \rightarrow m1n$$

Lines 94-101 declares an aggregate evaluator, here denoted $a1$, such that

$$a1: m1e \rightarrow LL3p$$

In other words, for each element, the aggregate evaluator provides a vector of 8 real numbers. The "Bind

Index" element on line 97 specifically indicates that the 8 entries of the vector are indexed by $LL3c$, which is the ensemble representing the 8 local nodes as was highlighted earlier, and so an alternative declaration would be:

$$a1: m1e \rightarrow LL3p$$

Which is equivalent to

$$a1: m1e \rightarrow (LL3c \rightarrow \mathbb{R})$$

(Note that the codomain of $a1$ is the signature of $v2$. By signature of a function, we mean the expression that appears after the colon when declaring the domain and codomain of a function.)

This means that $a1$ is a function that takes as input an element identifier, and returns a function. The function that it returns takes as input an index that identifies one of the 8 corners of the unit cube, and returns a real number that is the nodal value of the field at that corner.

Since the arrows in this notation are right-associative, this can also be written as

$$a1: m1e \to LL3c \to \mathbb{R}$$

Which can be viewed as $a1$ takes two parameters and returns a real number. This style is usually the result of a process known as "currying", where a function of multiple parameters is written as a function that returns a function, that may in turn also return a function, depending on the number of parameters. It is a common notational practice in lambda calculus and functional programming languages such as Haskell [38]. It is useful here, because it allows us to describe the behaviour of $a1$ as follows (by "uncurrying" the previous equation):

$$a1(e, n) = m1dof(m1c(e, n))$$

This is what the use of the "default" parameter on line 100 implies, in conjunction with the binding on lines 97 and 98. These indicate that the global node mapping, $m1c$, is used to map the element to the corresponding 8 global nodes.

Lines 106-112 declare a reference evaluator and perform binding of an existing evaluator, $LL3$, the tri-linear Lagrange interpolation basis function[34]. The reference evaluator under consideration defines a function $r1$, as follows:

$$r1: m1 \to \mathbb{R}$$

$$r1(e, \xi) = LL3(\xi, (n \mapsto a1(e, n)))$$

The definition of $r1$ implies that $\xi \in U3$, $e \in m1e$ and $\left(n \mapsto a1(e, n)\right) \in LL3p$.

(Also, the notation $n \mapsto a1(e, n)$ is the notation for a "lambda", which is just an anonymous function. For example, writing $f(x) = x^2$ is equivalent to writing $f = (x \mapsto x^2)$.)

Lines 120-125 define a piecewise evaluator, $m1pw$, which maps an element identifier to an interpolation function, but since all elements for all fields in this example use the same interpolation, it is equivalent to the "constant" $LL3$, meaning that the function $LL3$ is returned for any value of n.

$$m1pw: m1e \to (U3 \times LL3p \to \mathbb{R})$$

$$\forall e \in m1e, m1pw(e) = LL3$$

(The notation "$\forall e \in m1e$" reads "for all values of e that are in the set $m1e$".)

We will come back to the geometric coordinates field, and first describe the pressure field, which is slightly simpler, since it is a scalar field.

Lines 222-228 wrap the pressure DOFs, declaring pn such that

$$pn: m1n \to \mathbb{R}$$

Lines 188-218 are a data source for the pressure field DOFs, providing nodal values.

---

[34] Usually we speak of tri-linear Lagrange basis functions, i.e. in the plural. However, here it is thought of as a single function with just an additional parameter that picks out a particular function from the usual set of basis functions.

Lines 232-236 declare and define the interpolated pressure field, resulting in the declaration:

$$pr\colon (m1n \to \mathbb{R}) \to (m1 \to \mathbb{R})$$

Which is the same as saying

$$pr\colon (m1n \to \mathbb{R}) \to m1 \to \mathbb{R}$$

Its definition is

$$pr(pn, (e, \xi)) = s(\xi, l)$$

where

$$s = m1pw(e) = LL3$$

$$l = (n \mapsto pn(m1c(e, n)))$$

$$l \in LL3p$$

Coming back to the geometric coordinates, they are declared and defined in a similar way, except that now an additional piece of information is carried around to index into the three-dimensional coordinate vector.

Lines 130-160 provide DOF values for the 3D geometric coordinate field for each of the 20 mesh nodes.

Lines 165-172 wrap the geometric DOF values, declaring cn such that

$$cn\colon m1n \to \{1,2,3\} \to \mathbb{R}$$

Note that here $\{1,2,3\}$ is used to mean the set that indexes a vector in the standard library's declared $\mathbb{R}^3$.

Lines 179-185 declare the geometric coordinates using an aggregate evaluator gc such that

$$gc\colon (m1n \to \mathbb{R}) \to (m1 \to \mathbb{R}^3)$$

Note that since the codomain is a 3D vector, this is essentially equivalent to

$$gc\colon (m1n \to \mathbb{R}) \to m1 \to (\{1,2,3\} \to \mathbb{R})$$

which is equivalent to

$$gc\colon (m1n \to \mathbb{R}) \to m1 \to \{1,2,3\} \to \mathbb{R}$$

In other words, gc can be seen as a function that takes a DOFs source and mesh coordinates and returns a 3D vector value, or it can be seen as a function that takes a DOFs source, mesh coordinates and a value for the index of a 3D vector, and returns a real scalar. The key thing about an aggregate evaluator is that a different definition for computing can be used depending on the index. However, since all geometric coordinates (i.e. x, y and z) for all mesh elements in this example are interpolated the same way (using LLC), line 184 specifies that $m1pw$ is used in all cases. In other words:

$$\forall d \in \{1,2,3\},$$
$$gc(cn, (e, \xi), d) = s(\xi, l)$$

where

$$s = m1pw(e) = LL3$$

$$l = (n \mapsto cn(m1c(e, n), d))$$

$$l \in LL3p$$

Note that on line 179, the valueType attribute states that the codomain is $\mathbb{R}^3$. That is not the type, nor the codomain on $m1pw$, which is the default evaluator specified on line 184. Rather, $m1pw$ always evaluates to $LL3$, whose codomain is $\mathbb{R}$. So, this means that gc now requires the spatial coordinate to be specified, (i.e. 1, 2 or 3 for x, y or z), and only then does it satisfy the valueType attribute, thus the spatial coordinate identifier is treated as part of the function domain of gc. By contrast to $pr(pn, (e, \xi))$, which only requires pn and the pair (e, ξ), $gc(cn, (e, \xi), d)$ requires cn and the pair (e, ξ), but in addition, requires d, which identifies the spatial coordinate.

This illustrates the use of a key aspect of FieldML binding. Binding is permissive in the sense that allows new dependencies to be introduced into composition pipelines. This is a slight abuse of the standard notation $f: A \to B$. Usually it strictly indicates that f has domain A and codomain B. In FieldML, this is actually $f: (X1 \to A) \to (X2 \to B)$, where $X1$ and $X2$ are only known once binding is done. In other words, the domain and codomain of f depend on the context in which f is used. If f is bound so that its inputs are directly elements from A and its outputs are also directly elements of B, then $X1$ and $X2$ are known to be the nullary Cartesian product, written as just empty parenthesis, (). Anywhere that () appears as the domain of a function's signature, the signature may be collapsed to just the codomain. So, if $X1$ is (), then $X1 \to A = () \to A = A$. If either or both of $X1$ and $X2$ are not () then a new dependency has been introduced. For example, take $f: \{1,2\} \to \mathbb{R}$ such that

$$f(n) = \begin{cases} g(\xi)\ if\ n = 1 \\ h(\xi)\ if\ n = 2 \end{cases}$$

Using the above rule, this is actually $f: (X1 \to \{1,2\}) \to (X2 \to \mathbb{R})$, so here $X1 = ()$ and $X2 = \mathbb{R}$, so it is actually $f: \{1,2\} \to (\mathbb{R} \to \mathbb{R})$, which is the same as $f: \{1,2\} \to \mathbb{R} \to \mathbb{R}$. Uncurrying this makes it evident that binding has now given us an f that takes two parameters, i.e. it is now

$$f(n, \xi) = \begin{cases} g(\xi)\ if\ n = 1 \\ h(\xi)\ if\ n = 2 \end{cases}$$

Table 4 summarises the entire example.
