# Supplementary material for: FieldML, a proposed open standard for the Physiome project for mathematical model representation
Source: Med Biol Eng Comput. 2013 Jul 31;51(11):1191–207. doi: 10.1007/s11517-013-1097-7 (PMC3825639; doi:10.1007/s11517-013-1097-7)
Supplement: Supplementary file 3 — Supplementary material 3 (PDF 3236 kb) [file 11517_2013_1097_MOESM3_ESM.pdf]

Table 4 Equation style summary of line-by-line description of FieldML example XML

Line number	Object	Name	Equation	Description
13	Import: Ensemble Type	`trilinearLagrange.points`	$LL3c = \{1,2,\ldots,8\}$	Set of identifiers, essentially for the 8 corners of the unit cube
12	Import: Continuous Type	`trilinearLagrange.parameters`	$LL3p = \mathbb{R}^8$	8 real numbered nodal DOF values for the 8 corners of the unit cube for tri-linear Lagrange interpolation.
			$LL3p = (LL3c \to \mathbb{R})$	Alternate view of the above.
16	Import: Argument Evaluator	`trilinearLagrange.parameters.argument`		
19	Import: Argument Evaluator	`chart.3d.argument`	$\xi \in \mathbb{R}^3$	Unit cube
18	Import: External Evaluator	`trilinearLagrange.interpolator`	$LL3: \mathbb{R}^3 \times LL3p \to \mathbb{R}$	tri-linear Lagrange interpolation basis functions
21	Import: External Evaluator	`shape.unit.cube`	$pU3: \mathbb{R}^3 \to \mathbb{B}$	Shape predicate

25	Ensemble type	`mesh1.nodes`	$m1n = \{1,2,\dots,20\}$	
34	Argument Evaluator	`mesh1.nodes.argument`	$m1na \in m1n$	
38	Argument Evaluator	`mesh1.node.dofs.argument`	$m1dof: m1n \to \mathbb{R}$	
46	Mesh type's ensemble	`mesh1.mesh.type.elements`	$m1e = \{1, 2, \dots, 4\}$	Four elements in mesh
45	Mesh Type	`mesh1.mesh.type`	$m1 = m1e \times U3$	Four unit squares
54	Predicate		$U3 = [0,1]\times[0,1]\times[0,1]$	Unit square
83	Parameter Evaluator	`mesh1.trilinearLagrange.`<br>`connectivity`	$m1c: m1e \to LL3c \to m1n$	Local node to global node mapping. This is a matrix.
94	Aggregate Evaluator	`mesh1.trilinearLagrange.`<br>`parameters`	$a1: m1e \to LL3p$	Forms parameter vector of the 8 parameters required for tri-linear Lagrange interpolation of a scalar field for each element
			$a1: m1e \to (LL3c \to \mathbb{R})$	Alternative view of the above
			$a1: m1e \to LL3c \to \mathbb{R}$	Alternative view of the above, using currying style.
98, 100			$a1(e,n) = m1dof(m1c(e,n))$	Behaviour of the above: get global node from local to global node mapping,

				get DOF from nodal parameters for field.
106	Reference Evaluator	`mesh1.trilinear.interpolator`	$r1: m1 \to \mathbb{R}$<br>$r1(e,\xi) = LL3(\xi,(n \mapsto a1(e,n)))$	The definition of $r1$ implies that $\xi \in U3$, $e \in m1e$ and $a1(e) \in \mathbb{R}^8$.
120	Piecewise Evaluator	`mesh1.template.trilinear`	$m1pw: m1e \to (U3 \times LL3p \to \mathbb{R})$<br>$\forall e \in m1e, m1pw(e) = LL3$	
222	Parameter Evaluator	`mesh1.node.pressure`	$pn: m1n \to \mathbb{R}$	Pressure nodal DOFs
232	Reference Evaluator	`pressure`	$pr: (m1n \to \mathbb{R}) \to (m1 \to \mathbb{R})$<br>$pr: (m1n \to \mathbb{R}) \to m1 \to \mathbb{R}$	Pressure field declaration, equivalent forms.
			$pr(pn,(e,\xi)) = s(\xi,l)$<br>where<br>$s = m1pw(e) = LL3$<br>$l = (n \mapsto pn(m1c(e,n)))$<br>$l \in LL3p$	Pressure field definition
165	Parameter Evaluator	`mesh1.node.coordinates`	$cn: m1n \to \{1,2,3\} \to \mathbb{R}$	3D geometric coordinates DOFs. This is a matrix.

179	Aggregate Evaluator	`coordinates`	$gc: (m1n \to \mathbb{R}) \to (m1 \to \mathbb{R}^3)$<br>$gc: (m1n \to \mathbb{R}) \to m1 \to (\{1,2,3\} \to \mathbb{R})$<br>$gc: (m1n \to \mathbb{R}) \to m1 \to \{1,2,3\} \to \mathbb{R}$	Coordinates field declaration, equivalent forms.
			$\forall d \in \{1,2,3\},$<br>$gc(cn, (e, \xi), d) = s(\xi, l)$<br>where<br>$s = m1pw(e) = LL3$<br>$l = (n \mapsto cn(m1c(e, n), d))$<br>$l \in LL3p$	Coordinate field definition
